# Dynamic and flexible H3K9me3 bridging via HP1β dimerization establishes a plastic state of condensed chromatin

**DOI:** 10.1038/ncomms11310

**Published:** 2016-04-19

**Authors:** Kyoko Hiragami-Hamada, Szabolcs Soeroes, Miroslav Nikolov, Bryan Wilkins, Sarah Kreuz, Carol Chen, Inti A. De La Rosa-Velázquez, Hans Michael Zenn, Nils Kost, Wiebke Pohl, Aleksandar Chernev, Dirk Schwarzer, Thomas Jenuwein, Matthew Lorincz, Bastian Zimmermann, Peter Jomo Walla, Heinz Neumann, Tuncay Baubec, Henning Urlaub, Wolfgang Fischle

**Affiliations:** 1 Laboratory of Chromatin Biochemistry, Max Planck Institute for Biophysical Chemistry, Göttingen, Am Fassberg 11, 37077, Germany; 2 Bioanalytical Mass Spectrometry, Max Planck Institute for Biophysical Chemistry, Göttingen, Am Fassberg 11, 37077, Germany; 3 Applied Synthetic Biology, Institute for Microbiology and Genetics, Georg-August University Göttingen, 37077 Göttingen, Germany; 4 Department of Medical Genetics, Life Sciences Institute, The University of British Columbia, Vancouver, British Columbia, Canada V6T 1Z3; 5 Department of Epigenetics, Max Planck Institute of Immunobiology and Epigenetics, Stübeweg 51, 79108 Freiburg, Germany; 6 Biaffin GmbH & Co KG, Heinrich-Plett Strasse 40, 34132 Kassel, Germany; 7 Biomolecular Spectroscopy and Single-Molecule Detection, Max Planck Institute for Biophysical Chemistry, Göttingen, Am Fassberg 11, 37077, Germany; 8 Bioanalytics, Institute for Clinical Chemistry, University Medical Center Göttingen, Robert-Koch-Strasse 40, 37075 Göttingen, Germany; 9 Interfaculty Institute of Biochemistry, University of Tübingen, Hoppe-Seyler-Str. 4, 72076 Tübingen, Germany; 10 Department of Biophysical Chemistry, Technische Universität Braunschweig, Hans-Sommerstr. 10, 38106 Braunschweig, Germany; 11 Department of Molecular Mechanisms of Disease, University of Zürich, Winterthurerstrasse 190, 8057 Zürich, Switzerland

## Abstract

Histone H3 trimethylation of lysine 9 (H3K9me3) and proteins of the heterochromatin protein 1 (HP1) family are hallmarks of heterochromatin, a state of compacted DNA essential for genome stability and long-term transcriptional silencing. The mechanisms by which H3K9me3 and HP1 contribute to chromatin condensation have been speculative and controversial. Here we demonstrate that human HP1β is a prototypic HP1 protein exemplifying most basal chromatin binding and effects. These are caused by dimeric and dynamic interaction with highly enriched H3K9me3 and are modulated by various electrostatic interfaces. HP1β bridges condensed chromatin, which we postulate stabilizes the compacted state. In agreement, HP1β genome-wide localization follows H3K9me3-enrichment and artificial bridging of chromatin fibres is sufficient for maintaining cellular heterochromatic conformation. Overall, our findings define a fundamental mechanism for chromatin higher order structural changes caused by HP1 proteins, which might contribute to the plastic nature of condensed chromatin.

## Abstract (web summary)

Heterochromatin protein 1 (HP1), including HP1 α, β and γ, is a family of non-histone chromatin factors thought to be involved in chromatin organization. Here, the authors show that dimeric HP1β interacts dynamically with H3K9me3, a hallmark of heterochromatin, and bridges condensed chromatin.

## Graphical abstract

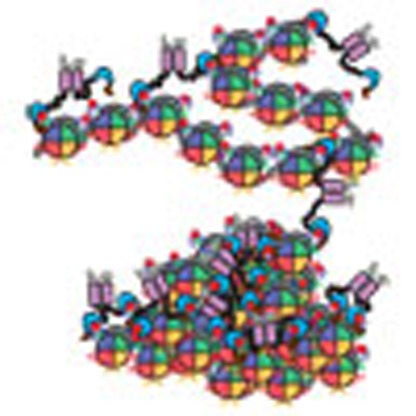

Heterochromatin is important for genome stability and transcriptional silencing through folding of chromatin into a condensed higher order structure. Methylation of lysine 9 within the histone H3 N-terminal tail is a crucial determinant of heterochromatin formation. The trimethylated form of this modification (H3K9me3) can be found at pericentric heterochromatin in virtually all higher eukaryotes and is viewed as a hallmark of transcriptionally silenced chromatin^1^,^2^.

Multiple evidence from genetic and cell biology studies points to an important involvement of heterochromatin protein 1 (HP1) factors, a family of non-histone chromatin proteins found in different isoforms in diverse organisms from *S. pombe* (Swi6) to human (HP1α, β and γ) in establishing and maintaining heterochromatic states^2^,^3^,^4^,^5^,^6^.

HP1 proteins generally contain two conserved globular domains, a chromo domain (CD) and a chromoshadow domain (CSD), which are linked by a less conserved, flexible hinge region (HR)^3^. Depending on the species and isoforms, additional less-conserved regions are found at the N and C termini of the proteins (NT and CT, respectively; Fig. 1a). The CD mediates interaction with H3K9me3 histone tail peptides with relatively low (micromolar) affinity^7^,^8^. Work on isolated chromatin components (peptides, histones, DNA) has suggested that the CD/H3K9me3 interaction might not be sufficient for chromatin targeting of HP1 proteins. Contacts via the HR or CSD might also be required. The issue is further complicated by self-dimerization of the CSD, which provides an interaction platform for a plethora of other proteins^9^,^10^. Several recent studies on human HP1α (hHP1α) using *in vitro* reconstituted ‘designer chromatin' containing defined histone modifications have reported conflicting results regarding binding specificity, chromatin effects as well as dependency on different domains of the protein^11^,^12^,^13^,^14^.

Here we investigated the molecular parameters and consequences of interaction between hHP1β and H3K9me3-containing oligonucleosomes. We focused on this isoform as it is the only essential mammalian HP1 protein, whose knockout in mice leads to perinatal lethality and severe genomic instability^15^. Our results demonstrate that mammalian HP1β is a prototypic HP1 protein, whose specific interaction with H3K9me3 chromatin only requires dimerization and spacing of the CD as well as general stabilization of this interaction by the NT. We suggest a general mechanism of dynamic higher order organization mediated by HP1 proteins that might be the basis of the plasticity of condensed chromatin.

## Results

### hHP1β binds H3K9me3 chromatin with high specificity

To obtain molecular insights into the interaction of HP1 proteins with chromatin, we generated uniformly K9-methylated histone H3 protein by native chemical ligation (synthetic H3 (aa 1–20) tail peptides fused to recombinant H3 (Δ1–20, A21C)) or via aminoalkylation of H3 with cysteine at position 9 instead of lysine (methyl lysine analogue, H3K_C_9me) using our previously established procedures^16^. Since our work has indicated sixfold reduced binding of hHP1β to an H3-tail peptide-containing H3K_C_9me compared with methylated lysine (Table 1)^17^, we used H3K_C_9me exclusively for experiments requiring large amounts of designer chromatin that were not available by the native chemical ligation strategy. H3 with different modification status was incorporated into mononucleosomes and 12-mer oligonucleosomal arrays using DNA containing 601 positioning sequences (Supplementary Fig. 1a,b). At saturation rates of 11±1 nucleosomes, we observed no apparent differences in the compaction and folding behaviour of the unmodified and H3K9me3 chromatin arrays (Supplementary Fig. 1c–e).

**Figure 1:**
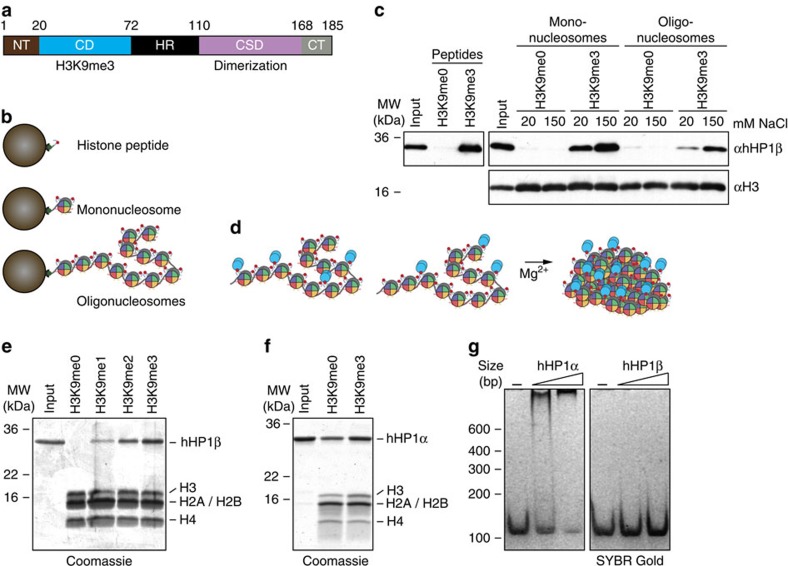
hHP1β–chromatin interaction is dependent on H3K9me3. (**a**) Domain structure of hHP1β; boundaries of domains are indicated by respective amino acid positions. For more details see Supplementary Fig. 2. (**b**) Scheme of pull-down experiments in **c** using different H3K9me templates immobilized on streptavidin-coated magnetic beads via C-terminal incorporation of a biotinylated lysine (peptide) or ligation of 5′-biotinylated oligonucleotides to DNA templates used in chromatin reconstitution (mono- and oligonucleosomes). (**c**) Immobilized H3K9me templates according to the pull-down experimental schemes in **b** were incubated with recombinant hHP1β WT. Material recovered after washing was analysed by western blotting. The indicated salt concentrations were used throughout the experiment. (**d**) Scheme of chromatin coprecipitation assay; factors bound to oligonucleosomes are precipitated with the template when clustering is induced by addition of Mg^2+^ ions. (**e,f**) Chromatin coprecipitation of hHP1β (**e**) and hHP1α (**f**) proteins with oligonucleosomes. Precipitated material was run on SDS-PAGE and stained with Coomassie blue. Input, 10%. (**g**) The indicated recombinant proteins were incubated with a DNA fragment of 150 bp at 500: 1 and 1,000: 1 molar ratio. Complexes were separated by PAGE. DNA was stained with SYBR Gold.

In pull-down experiments of immobilized H3 tail peptides, mono- and oligonucleosomes hHP1β was retained much more efficiently on all three matrices in the presence of H3K9me3 as compared with the unmodified (H3K9me0) counterpart (Fig. 1b,c; Supplementary Table 1 summarizes the different assays and experimental conditions used to analyse hHP1β/chromatin interaction). Since chromatin fibres undergo reversible transitions from elongated state to compacted arrays that are further aggregated by interstrand interaction^18^, we asked whether specific binding was retained with oligonucleosomes at higher order folded state. In the presence of 5 mM Mg^2+^, chromatin fibres precipitate and can be recovered from solution by centrifugation. Under these conditions hHP1β coprecipitated only with H3K9me1/2/3 but not H3K9me0 oligonucleosomes (Fig. 1d,e). Further analysis with H3K4me3 and H3K_C_27me3 oligonucleosomes verified the specificity of this interaction (Supplementary Fig. 1f and g). Recovery of hHP1β with aggregated chromatin was independent of the DNA sequence used for chromatin reconstitution (Supplementary Fig. 1h).

When analysing recombinant hHP1α in the chromatin coprecipitation scheme, the protein displayed much less specificity for H3K9me3 compared with hHP1β (Fig. 1f). In agreement and as observed previously, hHP1α but not hHP1β showed a high level of general DNA binding in gel shift experiments (Fig. 1g)^13^.

### Dimerization mediates H3K9me3-chromatin binding of hHP1β

**Figure 2:**
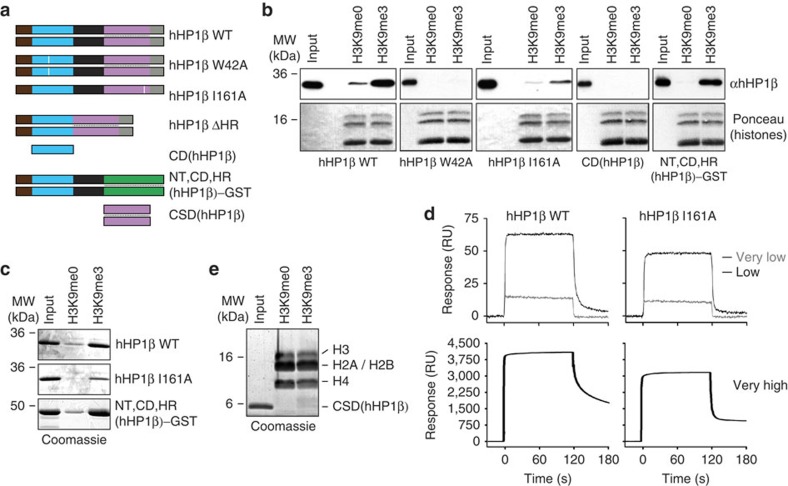
Dimerization is necessary and sufficient for hHP1β binding of H3K9me3 chromatin. (**a**) Schematic representation of hHP1β mutant proteins. White bars indicate positions of W42 and I161, respectively. (**b**) Chromatin coprecipitation of WT and mutant hHP1β proteins with oligonucleosomes. Precipitated material was analysed by western blotting using antibodies that recognizes the CD of hHP1β. Ponceau staining of the region of the western blot membrane containing histones is shown as loading control. Input, 10%. (**c**) The indicated proteins were incubated with a biotinylated H3K9me3 peptide immobilized on magnetic streptavidin beads. Material recovered after washing was run on SDS–PAGE and stained with Coomassie blue. Input, 10%. (**d**) SPR analysis of hHP1β WT interaction with a biotinylated H3K9me3 peptide immobilized at different density on the chip surface (very low, 5 RU; low, 24 RU; very high, 950 RU). (**e**) Experiment as in **b** using hHP1β CSD, but analyzed by SDS–PAGE and staining with Coomassie Blue.

To determine the molecular requirements for the highly specific hHP1β/H3K9me3–chromatin interaction, we prepared different point as well as deletion mutant proteins (Fig. 2a and Supplementary Fig. 2a). Mutation of residue W42, which was shown previously to impair binding to H3K9me3 (ref. 7), resulted in the loss of recovery of hHP1β with H3K9me3 oligonucleosomes (Fig. 2b). Under the same conditions neither the monomeric CD alone nor an I161A mutant protein, which is incapable of dimerization^8^, were significantly recovered with H3K9me3 oligonucleosomes. Since both proteins bound an H3K9me3-peptide as efficiently as hHP1β wild type (WT; Table 1), the results implied that the CD/H3K9me3 interaction is not sufficient for chromatin binding. In agreement, hHP1β I161A was ineffective in competing with hHP1β WT for interaction with H3K9me3 oligonucleosomes (Supplementary Fig. 2b). Also, isothermal titration calorimetry (ITC) measurements deduced a 12-fold reduced interaction of hHP1β I161A with H3K_C_9me3 oligonucleosomes compared with hHP1β WT (Table 1).

We reasoned that the difference in binding of hHP1β WT and I161A proteins to free peptide and oligonucleosomes must be due to the high density of H3K9me3 on the chromatin template. Indeed, pull-down experiments with H3-tail peptides densely immobilized on magnetic beads showed significantly reduced recovery of I161A compared with hHP1β WT (Fig. 2c).

Further analysis of the interaction kinetics of hHP1β WT and I161A with H3K9me3 peptides immobilized on surface plasmon resonance (SPR) chip surfaces deduced similar, very fast association kinetics of both proteins. The signal of immobilization rapidly reached a plateau with both proteins and on H3K9me3 surfaces of different density (Fig. 2d). The hHP1β WT and I161A proteins differed, however, in the release from the matrices. While the I161A protein dissociated rapidly from the H3K9me3 ligand, hHP1β WT was released more slowly. This effect was more pronounced at higher H3K9me3 surface densities. The residual signals seen under these conditions were lower at higher flow rates and could also be competed specifically with free H3K9me3 peptide ligand but not H3K9me0 (Supplementary Fig. 2c and d), indicating that this effect is due to mass transfer limitations as well as rebinding phenomena known to occur with SPR measurements^19^. In agreement with the different binding kinetics, we deduced 10-fold stronger binding of the hHP1β WT protein to the H3K9me3 matrix compared with the I161A protein in SPR titration experiments (Table 1).

Since we did not detect any binding of the CSD to oligonucleosomes (Fig. 2e), we reasoned that its contribution to H3K9me3/chromatin interaction is solely via mediating dimerization. To test this hypothesis, we analysed an hHP1β chimera where the CSD was replaced with an unrelated dimerization module. We used glutathione S-transferase (GST), as it is known to form dimers of similar strength as hHP1β WT in solution (*K*_d_<1 nM)^9^,^20^. In chromatin coprecipitation (Fig. 2b) as well as in H3K9me3 peptide pull-down (Fig. 2c) experiments NT, CD, HR(hHP1β)-GST indeed showed interaction similar to hHP1β WT. On the basis of these findings, we deduced that CSD-mediated dimerization of hHP1β WT results in kinetic trapping of the protein on dense H3K9me3 surfaces such as provided on peptide-bound matrices or oligonucleosomes and there in particular after condensation and aggregation.

### hHP1β induces aggregation of H3K9me3 oligonucleosomes

**Figure 3:**
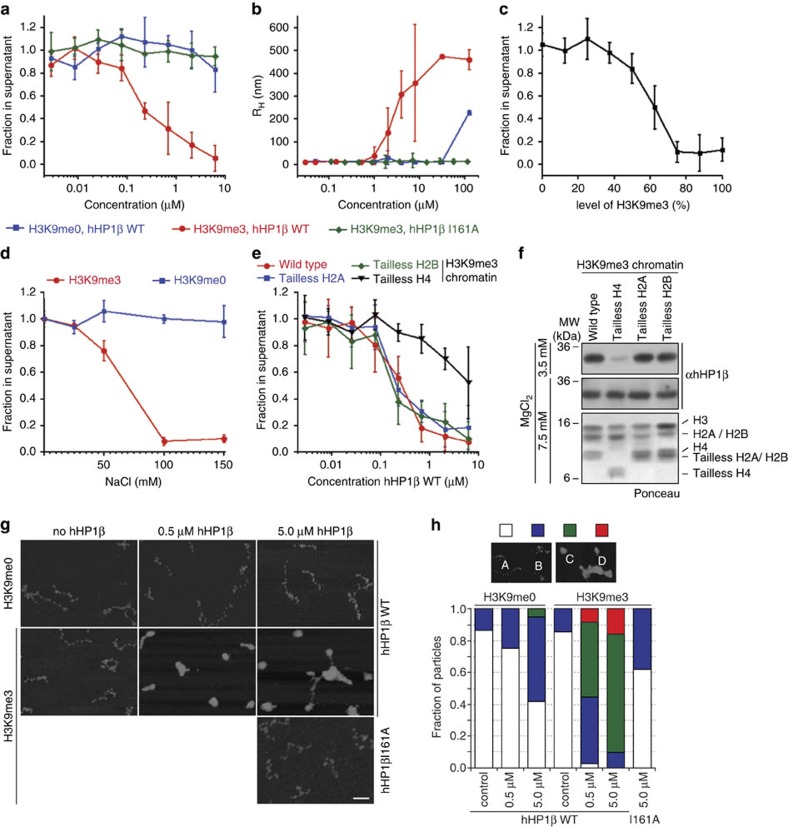
hHP1β clusters oligonuclesomal arrays in dependence of H3K9me3. (**a**) Oligonucleosomes (2.5 nM) were incubated with increasing concentrations of hHP1β WT or I161A at 150 mM NaCl. DNA remaining in the supernatant after centrifugation was incubated with EtBr and measured by fluorescence reading. Data were normalized to amounts in absence of added protein. Averages of three independent experiments are plotted; error bars represent s.d.; *n*=3. (**b**) Analysis of the hydrodynamic radius of oligonucleosomes (10 nM; DNA labelled with ATTO 610) in the presence of increasing concentrations of hHP1β WT or I161A by FCS at 150 mM NaCl. For details on experimental setup see Supplementary Fig. 3. Error bars represent s.d.; n=3. (**c**) Oligonucleosomes were reconstituted after mixing H3K9me0 and H3K9me3 octamers at different ratio. Chromatin precipitation analysis at 150 mM NaCl was carried out with hHP1β WT at saturating concentration. Data are presented as in **a**. (**d**) Precipitation behaviour of oligonucleosomes (2.5 nM) was analysed at different concentrations of NaCl with hHP1β WT at saturating concentration. Data are presented as in **a**. (**e**) H3K9me3-containing chromatin was reconstituted using wild-type core histones or tailless H2A, H2B or H4. Oligonucleosomes (2.5 nM) were incubated with increasing concentrations of hHP1β WT at 150 mM NaCl. Data are presented as in **a**. (**f**) Chromatin coprecipitation of hHP1β with H3K9me3-containing oligonucleosomes reconstituted with wild-type or tailless core histones at the indicated concentrations of MgCl_2_. Precipitated material was analysed by western blotting. The region of the western blot membrane of the experiment at 7.5 mM MgCl_2_ containing histones was stained with Ponceau. (**g**) Oligonucleosomes at 50 mM NaCl were incubated with hHP1β WT or I161A. Complexes were fixed with 0.05% (v/v) glutaraldehyde, spotted on mica surfaces and analysed by scanning force microscopy. Control, no protein added; scale bar, 100 nm. (**h**) Quantification of results representatively shown in **g** according to the classification on the top: *A*, extended; *B*, partially condensed; *C*, fully condensed; *D*; aggregates; *n*>50 for each condition.

Having established the interaction parameters of hHP1β with oligonucleosomes, we investigated the effects of its binding onto chromatin conformation. At 150 mM NaCl oligonucleosomes are highly soluble. These are in compacted (zig-zag) state but do not interact with each other (Supplementary Fig. 1e). Under these conditions increasing concentrations of hHP1β WT efficiently aggregated H3K9me3 but not H3K9me0 oligonucleosomes to a degree that the complexes precipitated and could be collected by centrifugation (Fig. 3a). This effect was generally reversible as shown in resolubilization experiments (Supplementary Fig. 3a and b). The hHP1β I161A mutant protein, in contrast, failed to induce the aggregation of oligonucleosomes.

To obtain further insights into the chromatin aggregation induced by hHP1β, we set up fluorescence correlation spectroscopy (FCS) measurements for single-molecule analysis. By labelling the DNA in oligonucleosomes with ATTO 610, this allowed delineating the diffusion behaviour and thereby size of the different chromatin species (Supplementary Fig. 3c and d). In this assay, hHP1β WT induced H3K9me3 oligonucleosomes to form large assemblies that reached a maximum hydrodynamic radius of 450 nm at the highest protein concentration (Fig. 3b). No such complex formation by clustering was seen with the I161A mutant protein. With the H3K9me0 oligonuclesomes larger assemblies only appeared at high hHP1β WT concentrations. We note that the half-maximal effects in the FCS experiment and the chromatin precipitation assay were seen at 3.5 and 0.4 μM hHP1β WT, respectively. Since the binding of hHP1β to peptides is slightly enhanced at lower temperature^21^, we assume that the difference is explained by the experimental setup of the FCS experiment at 23°C and the sedimentation assay at 4°C. Also, we do not know the minimal hydrodynamic size of aggregates that results in the recovery in the centrifugation assay.

### hHP1β clusters chromatin exposing high density of H3K9me3

In analysing the interaction of hHP1β WT with H3K9me3 in different biochemical context, we noticed a significant difference in binding strength at low and high-salt concentrations. While the protein bound better to free H3K9me3 peptide at 20 mM NaCl (*K*_d_=1.4±0.6 μM) compared with 150 mM NaCl (*K*_d_=3.3±0.2 μM), this trend was reversed in the context of chromatin. H3K_C_9me3 mononucleosomes were bound 2.7-fold better at the higher compared with the lower salt concentration. At 20 mM NaCl we could not detect any binding of hHP1β WT to H3K_C_9me3 oligonucleosomes but deduced a *K*_d_ of 11±2 μM for binding to the same template at 150 mM NaCl (Table 1 and Supplementary Fig. 4). Obviously, two counteracting effects determine binding of hHP1β to H3K9me3 chromatin. On one end, the CD/H3K9me3 interaction is sensitive to higher salt conditions. On the other end, at low salt concentration, the H3 tails in chromatin are not fully available, likely due to electrostatic binding to DNA^18^,^22^. Conditions of higher salt increase the availability of the H3 tails and induce chromatin condensation. The high density of H3K9me3 in this situation provides a favourable binding platform for hHP1β interaction, which in turn promotes clustering of chromatin.

If this interpretation were correct, we would expect the effect of hHP1β chromatin clustering to be dependent on the level of H3K9me3 in the oligonucleosomes. Indeed, when mixing H3K9me0 and H3K9me3 at different ratios for oligonucleosomal reconstitution, we observed that a content of more than 50% H3K9me3 was required for hHP1β to induce efficient chromatin clustering (Fig. 3c).

To further test the idea that hHP1β preferentially binds to condensed chromatin and mediates clustering, we performed salt titration of H3K9me0 and H3K9me3 oligonucleosomes in presence of the protein. We found a transition of hHP1β inducing clustering of H3K9me3 oligonucleosomes at salt concentrations above 50 mM NaCl, which are known to promote intrafiber condensation (Fig. 3d). No clustering of H3K9me0 chromatin was seen in the presence of hHP1β even at 150 mM NaCl.

Mg^2+^-induced chromatin aggregation requires minimally the N-terminal tails of H3 or H4. In contrast, the tails of H2A and H2B are not sufficient for this process^23^,^24^. To further analyse hHP1β-dependent chromatin clustering, we set up H3K9me3 oligonucleosomal templates containing tailless H2A, H2B or H4. Compared with oligonucleosomes containing WT histones these required higher MgCl_2_ concentration for inducing intrafiber aggregation and precipitation (Supplementary Fig. 5), as was shown previously^24^. We determined the Mg50 value for WT H3K9me3 oligonucleosomes at around 2 mM, whereas the Mg50 for H3K9me3 oligonucleosomes containing tailless H2A or H2B were around 3 mM and that for H3K9me3 oligonucleosomes containing tailless H4 was around 4 mM. While hHP1β clustered H3K9me3 oligonucleosomes containing tailless H2A or H2B as efficiently as H3K9me3-oligonucleosomes containing WT core histones, the effect on H3K9me3 oligonucleosomes containing tailless H4 was impaired (Fig. 3e). Also and in agreement with the altered aggregation behaviour (Supplementary Fig. 5), chromatin coprecipitation showed reduced recovery of hHP1β with H3K9me3 oligonucleosomes in the absence of the H4 N-terminal tail at 3.5 mM MgCl_2_, whereas recovery at 7.0 mM MgCl_2_ was comparable to the level obtained with H3K9me3 oligonucleosomes containing WT core histones (Fig. 3f).

Since the salt titration had shown that at 50 mM NaCl discriminatory effects of hHP1β onto H3K9me0 and H3K9me3 oligonucleosomes become apparent (Fig. 3d), we selected this condition to visualize the chromatin complexes formed by hHP1β using scanning force microscopy (Fig. 3g). Due to the clear dose response (Fig. 3a,b), we reasoned that at this intermediate condition different states of hHP1β-dependent chromatin clustering could be observed at different concentrations of the protein. At 0.5 μM hHP1β WT, we mostly observed structures whose size was consistent with individual but highly condensed H3K9me3 chromatin species. At 5 μM hHP1β WT larger clusters that must contain multiple oligonucleosomes were visible. Similar to the ‘in solution' assays, the frequency of condensed chromatin fibres and large chromatin clusters was much lower for H3K9me0 chromatin with hHP1β WT or for H3K9me3 chromatin with I161A mutant protein (Fig. 3h).

### hHP1β bridges H3K9me3 of different chromatin fibres

**Figure 4:**
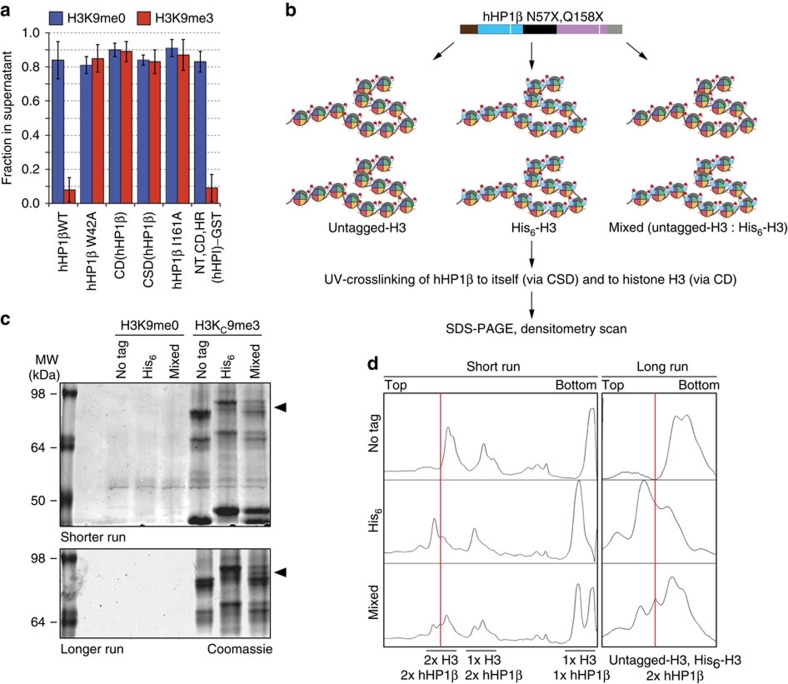
hHP1β bridges nucleosomes on the same and different chromatin fibres. (**a**) Chromatin precipitation analysis of oligonucleosomes with hHP1β wild-type (WT) or mutant proteins under saturating conditions. DNA remaining in the supernatant after centrifugation was incubated with EtBr and measured by fluorescence reading. Data were normalized to amounts present in the input. Error bars represent s.d.; n=3. (**b**) Scheme of UV-mediated hHP1β-histone H3 cross-linking experiments. (**c**) Photo cross-linking according to the scheme in **b** was done with H3K9me0 or H3K_C_9me3 oligonucleosomal arrays and hHP1β N57X Q158X. Samples were run on SDS–PAGE (top, shorter run; bottom, longer run) and stained with Coomassie blue. Black arrows indicate crosslinked hHP1β dimer crosslinked to one untagged histone H3 and one His_6_-tagged histone H3. (**d**) Histograms of intensity scans of Coomassie blue stained SDS–PAGE gels shown in **c**.

We next investigated whether dimerization is sufficient for the effect of the hHP1β CSD in chromatin clustering. At 150 mM NaCl 5 μM hHP1β WT are sufficient for inducing maximal chromatin clustering (Fig. 3a). Under these conditions, neither the I161A mutant, nor the isolated CD or CSD had any effect on modified or unmodified chromatin (Fig. 4a). As expected, mutation of W42A that abolishes interaction with H3K9me3 also failed to cluster oligonucleosomes. However, artificial dimerization of the NT, CD and HR domains of hHP1β via GST (NT, CD, HR(hHP1β)-GST) was sufficient to induce the maximal response in this assay.

To determine whether hHP1β promotes interfiber association by bridging different chromatin strands, we set up a cross-linking strategy, where we mapped linkage of dimeric protein to H3 (Fig. 4b). The photo-crosslinkable unnatural amino acid *p*-benzoyl-L-phenylalanine (pBpa) covalently binds to aliphatic side chains of amino acids within a distance of <4 Å on ultraviolet irradiation (*λ*=365 nm). On the basis of the available three-dimensional structures of CD/H3K9me3 and CSD/CSD complexes, we used stop codon suppression to generate a recombinant hHP1β protein containing pBpa at positions 57 and 158 (N57X and Q158X) for cross-linking of the CD and CSD, respectively (Supplementary Fig. 6a)^25^. Incorporation of pBpa at these sites did not affect the binding of hHP1β to H3K9me3 oligonucleosomal arrays (Supplementary Fig. 6b). To distinguish H3 from different chromatin fibres, we reconstituted oligonucleosomes with untagged and His_6_-tagged H3 unmodified and containing the H3K_C_9me3 methyl lysine analogue (Fig. 4b). Chromatin coprecipitation analysis verified that the His_6_-tagging of H3 did not have any effect on hHP1β recruitment (Supplementary Fig. 6c).

While hHP1β N57X Q158X did not cross-link to H3 in unmodified oligonucleosomes (Fig. 4c, Supplementary Fig. 6d shows the loading control of the experiment), western blotting with anti-His_6_ and anti-H3 antibodies identified three major crosslinks of hHP1β N57X Q158X to H3K_C_9me3 oligonucleosomes under conditions of chromatin clustering (Supplementary Fig. 6e): 1 × H3 crosslinked to 1 × hHP1β monomer, 1 × H3 crosslinked to 1 × hHP1β dimer and 2 × H3 crosslinked to 1 × hHP1β dimer. Importantly, with an equimolar mixture of untagged and His_6_-tagged H3K_C_9me3 oligonucleosomes crosslink of an hHP1β N57X Q158X dimer to both H3 species was observed (Fig. 4c,d). Further analysis of the hHP1β I161A and NT, CD, HR(hHP1β)-GST mutant proteins containing the pBpa only at positions 57 within the CD verified that dimerization is absolutely required for cross-linking to H3 in this scheme (Supplementary Fig. 6f).

### HR and NT of hHP1β modulate the function of CD and CSD

To obtain further insights into the binding interfaces of hHP1β with H3K9me3 chromatin, we set up cross-linking experiments that were analysed by mass spectrometry. In particular, we targeted electrostatic protein–protein interactions using 1-Ethyl-3-[dimethylaminopropyl]carbodimine (EDC), which is a zero length amine-carboxyl crosslinker (Supplementary Data 1 contains a summary of all crosslinks identified). In free hHP1β a large number of crosslinks originating from all domains of the protein and connecting to other regions of the factor were identified (Supplementary Fig. 7a). The results were in agreement with an overall high flexibility of the protein as determined by SAXS and nuclear magnetic resonance measurements^16^.

**Figure 5:**
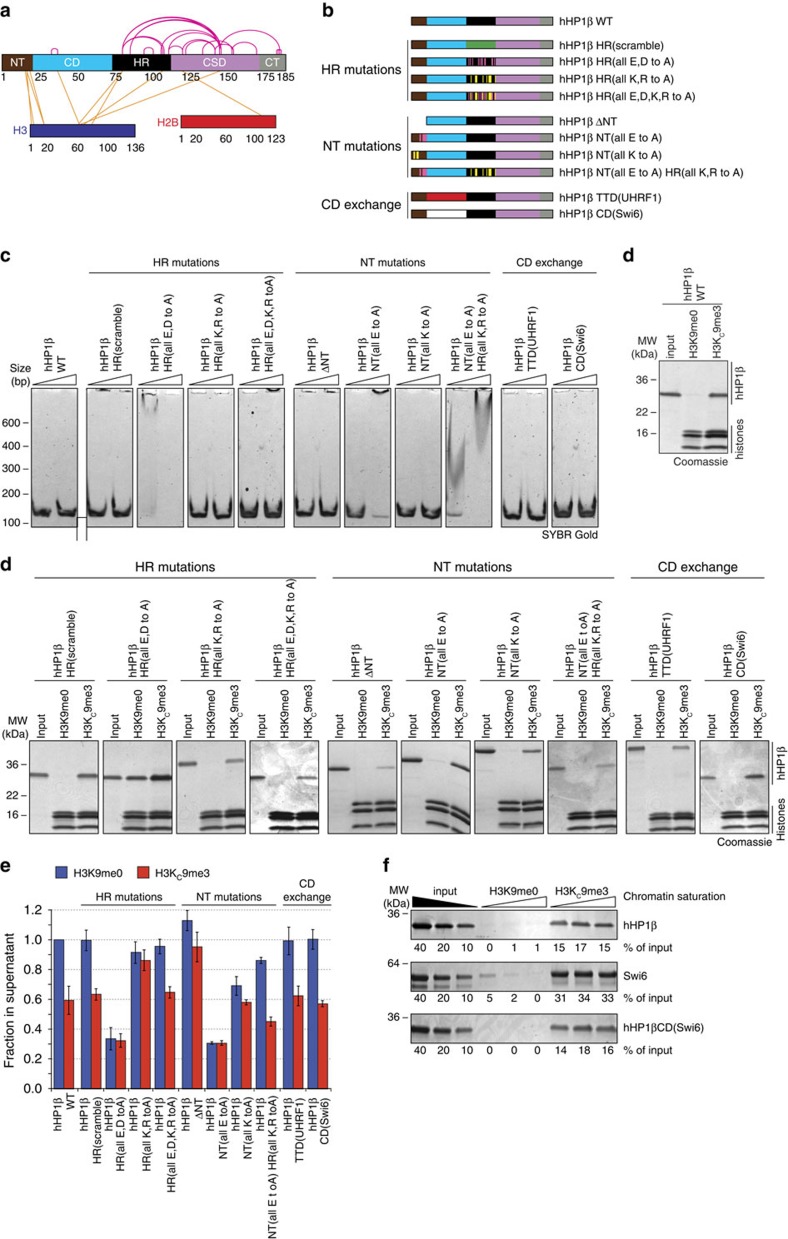
Modulation of CD and CSD functions by the HR and NT of hHP1β. (**a**) Scheme representing EDC cross-links identified within the hHP1β/H3K_C_9me3 oligonucleosome complex under conditions of chromatin clustering (13.4 nM H3K_C_9me3 oligonucleosomal arrays, 15 μM hHP1β, 100 mM NaCl) using mass spectrometry. For detailed listing of the crosslinks see Supplementary Data 1. (**b**) Schematic representation of hHP1β mutant proteins. (**c**) The indicated recombinant proteins were incubated with a DNA fragment of 150 bp at 500: 1 and 1,500: 1 molar ratio. Complexes were separated by PAGE. DNA was stained with SYBR Gold. (**d**) Chromatin coprecipitation of the indicated wild-type (WT) and mutant hHP1β proteins with oligonucleosomes. Precipitated material was run on SDS–PAGE and stained with Coomassie blue. Input, 10%. (**e**) Chromatin precipitation analysis of oligonucleosomes with hHP1β WT or mutant proteins under non-saturating conditions. DNA remaining in the supernatant after centrifugation was incubated with EtBr and measured by fluorescence reading. Data were normalized to DNA levels present in the H3K9me0 chromatin/hHP1β WT sample. Averages of three independent experiments are plotted; error bars represent s.d.; *n*=3. (**f**) Chromatin coprecipitation of the indicated proteins with oligonucleosomes reconstituted to different saturation (0.8: 1.0, 1.0: 1.0 and 1.0: 1.2 ratio of octamers to positioning sequences). Precipitated material was run on SDS–PAGE and stained with Coomassie blue. Intensity of bands was quantified in relation to the input. Representative experiment is shown.

Far fewer crosslinks within hHP1β in the presence of H3K9me3 oligonucleosomes under conditions of chromatin clustering (Fig. 5a) and chromatin coprecipitation (Supplementary Fig. 7b) implied less flexibility of the protein in the bound state. In particular, all crosslinks involving the CD were lost. Also, crosslinks within the NT and from the NT to the CSD were suppressed. In contrast, a number of interfaces between residues in the HR and CSD remained as in the free state. While very few contacts with chromatin were found for H2B and H4, by far the most connections of different regions of hHP1β were with H3. Here especially residues in the NT were found in many crosslinks to the N-terminal tail of H3 proximal and distal to the K9 site. The CSD and CT also had some contacts in this area and CD, HR and CSD were found linked to few sites within the H3 core region (aa 60–80).

To further investigate these findings, we established a series of hHP1β proteins mutated within the NT and HR (Fig. 5b). None of these mutant proteins differed significantly from hHP1β WT in their binding to a free H3K9me3 tail peptide (Supplementary Fig. 7c). To detect a wider range of effects, we refined the chromatin clustering assay using the mutant hHP1β proteins at non-saturating level and lowering the NaCl concentration to 100 mM. Randomizing the sequence of the HR (hHP1β HR(scramble)) had no effect on the ability of hHP1β to cluster chromatin. Since work on HP1α proteins has implied this region in DNA/RNA binding^26^,^27^,^28^, we also eliminated all the negative (hHP1β HR (all E, D to A)) or positively charged residues (hHP1β HR (all K, R to A)) separately as well as altogether (hHP1β HR (all E,D,K,R to A); Supplementary Table 2). In agreement with strong, unspecific DNA binding (Fig. 5c) the hHP1β HR (all E, D to A) mutant was recovered much more efficiently on H3K9me3 chromatin compared with the WT protein but also showed high recovery on the H3K9me0 template (Fig. 5d). It had very strong chromatin clustering activity and precipitated H3K9me0 and H3K9me3 oligonucleosomes equally well (Fig. 5e). In stark contrast, the hHP1β HR (all K, R to A) mutant protein, which did not bind DNA, was less recovered with the H3K9me3 template and lost its ability to cluster chromatin. Neutralizing all charges of the HR in the hHP1β HR (all E, D, K, R to A) mutant not only recapitulated the lack of DNA binding of the WT protein but also fully restored its activity in chromatin coprecipitation and clustering.

On the basis of these results, we concluded that the HR of hHP1β does not directly contribute to chromatin binding and clustering but provides a flexible and spacing linker. Increase of the net negative charge of this region results in repelling of DNA, whereas increase of the net positive charge causes unspecific chromatin binding. In agreement with this interpretation, deletion of the HR resulted not only in significant reduction of the chromatin clustering activity of hHP1β but also of the NT, CD(hHP1β)-GST hybrid protein (Supplementary Fig. 7d and e). Furthermore, exchange of the hHP1β HR with the corresponding regions of hHP1α and Swi6, which contain a surplus of positive charges (Supplementary Fig. 2a and Supplementary Table 2), caused strong DNA binding concomitant with strong chromatin clustering of both, H3K9me0 and H3K9me3 oligonucleosomes (Supplementary Fig. 7f and g).

The NT region of hHP1β is also highly enriched in charged amino acids (Supplementary Fig. 2a). In agreement with the manifold contacts with the H3 tail detected in the cross-linking experiments, deletion of this region (hHP1β ΔNT) caused loss of recovery with precipitated H3K9me3 chromatin (Fig. 5d) as well as failure of chromatin clustering (Fig. 5e). Mutating the negatively charged residues (hHP1β NT (all E to A)) enhanced DNA binding and resulted in strong, unspecific chromatin clustering. Mutating the positively charged residues (hHP1β NT (all K to A)) had no effect on DNA binding but caused enhanced chromatin clustering of H3K9me0, while the effect on H3K9me3 oligonucleosomes was similar to hHP1β WT. Together with the results from the cross-linking, we concluded that the NT of hHP1β balances unspecific binding to DNA and the H3 tail. Indeed, opposing double mutation of charged residues in the NT and HR regions neutralized each other's chromatin effects (hHP1β NT (all E to A) HR (all K,R to A), Fig. 5d,e).

### CD of hHP1β only functions in mediating H3K9me3 interaction

In contrast to what has been described for Swi6 (ref. 29), we failed to detect any multimerization of hHP1β beyond CSD-mediated dimerization using highly sensitive dynamic light scattering (Supplementary Fig. 8a), SAXS^16^, protein cross-linking (Supplementary Fig. 7a) or nuclear magnetic resonance^30^. Furthermore, SPR experiments similar to those done with Swi6 on H3K9me3 peptide and H3K9me3 mononucleosomes coated surfaces were fully consistent with a bivalent interaction mode of the hHP1β dimer but did not point to any preformed or induced higher order multimerization of the protein (Supplementary Fig. 8 and Supplementary Note 1).

To address this discrepancy, we compared the chromatin binding and effects of hHP1β and Swi6. hHP1β bound specifically to H3K9me3 oligonucleosomes irrespective of the saturation level of the chromatin template with nucleosomes (Fig. 5f and Supplementary Fig. 9a). In contrast, the specificity of Swi6 association with H3K9me0 and H3K9me3 oligonucleosomes increased significantly when the chromatin was more saturated. This effect is caused by the strong unspecific DNA binding activity of the HR of Swi6 (Supplementary Fig. 9b). Interestingly, we found Swi6 recovered to a higher degree in H3K9me3 chromatin coprecipitation compared with hHP1β. Quantification deduced a stoichiometry of approximately four Swi6 molecules but only two hHP1β molecules bound per nucleosome under saturating protein concentrations (Fig. 5f and Supplementary Fig. 9c).

While it had been suggested before that CD–CD interactions mediate tetrameric binding of Swi6 on mononucleosomes^29^, this region was clearly not sufficient for the observed effect. A hybrid hHP1β protein where the CD was replaced with that of Swi6 (hHP1β CD(Swi6)) did behave like hHP1β WT, not like Swi6. It also exhibited a chromatin clustering effect equivalent to hHP1β WT (Fig. 5d,e). Obviously, the additional effects of Swi6 must reside in regions outside the CD. We deduced that the only role of the CD in HP1 chromatin binding is indeed in mediating targeting to H3K9me3. To further sustain this idea, we replaced the CD of hHP1β with the tandem tudor domain (TTD) of UHRF1, which has been shown to specifically recognize H3K9me3 with micromolar affinity (Supplementary Fig. 7c)^31^. Indeed, the hybrid hHP1β TTD(UHRF1) protein displayed chromatin binding and clustering reminiscent of hHP1β WT (Fig. 5d,e).

### mHP1β localizes to genomic regions enriched for H3K9me3

Having established the basic molecular parameters of hHP1β chromatin binding and effects *in vitro*, we wanted to determine whether these are applicable in a cellular context. Our results predicted that HP1β binds more stably to chromatin areas where the local concentration of H3K9me3 is high. In absence of methods to detect three-dimensional enrichment of H3K9me3 in defined subnuclear volumes, we looked at the distribution of HP1β along the genome (that is, localization in one dimension). We employed genome-wide binding analysis of HP1β in mouse embryonic stem cells (mESC). The sequences of mouse and human HP1β proteins are identical and mESC with knock out of essential H3K9me3 methyltransferases exist^32^.

**Figure 6:**
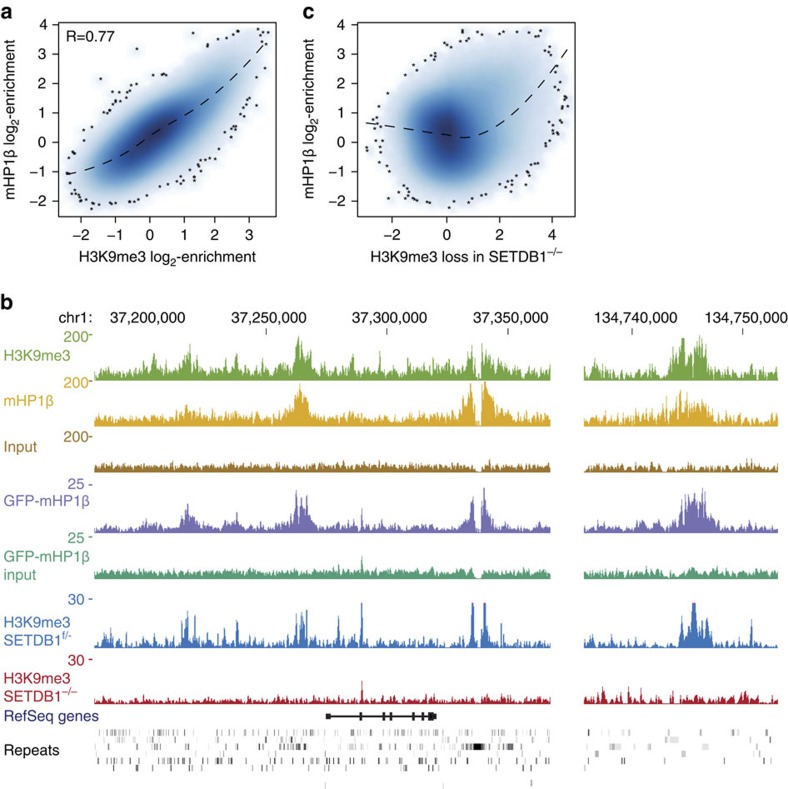
Genome-wide enrichment of HP1β correlates with SETDB1-dependent H3K9me3 deposition. (**a**) Plot of genome-wide correlation of mHP1β and H3K9me3 (ref. 35) as deduced by ChIP-seq in mouse ESC and using 1 kb-sized windows. Dashed line indicates the data trend computed by loss regression. (**b**) Tracks displaying number of library-normalized reads per 100 bp from the indicated ChIP-seq experiments. Data obtained from antibody-based enrichments of endogenous mHP1β (top) and GFP-tagged mHP1β (middle) and the respective input tracks are shown. H3K9me3 data in SETDB1^f/−^ and SETDB1^−/−^ experiments are from ref. 36. Gene models and repetitive elements are indicated at the bottom. (**c**) Analysis as in **a** but comparing mHP1β binding to H3K9me3 loss in SETDB1^−/−^ mESC. H3K9me3 loss is shown as the log_2_-difference between SETDB1^f/−^ and SETDB1^−/−^.

Previous genome-wide analysis mainly focused on correlations between mHP1α and H3K9me3 at repetitive elements^33^. Given the general high abundance of H3K9me3 at these regions^34^, this does not allow obtaining quantitative estimation of HP1 dependency on genome-wide H3K9me3 levels. To address this, we performed genome-wide antibody-based chromatin immunoprecipitation-sequencing (ChIP-seq) to measure mHP1β binding and compared the results with H3K9me3 enrichment previously obtained in the same mESC line^35^. The mouse genome was partitioned into 1 kb-sized tiles, and mHP1β and H3K9me3 enrichments above input were calculated for each of these tiles. mHP1β and H3K9me3 were found highly correlated along the entire genome (Fig. 6a, Pearson's *R*=0.77), and this was also evident at single loci (Fig. 6b). Importantly, an independent data set consisting of green fluorescent protein (GFP)-tagged mHP1β that was generated in a separate mESC line confirmed these findings (Supplementary Fig. 10a and b). Furthermore, by measuring genome-wide changes in H3K9me3 after conditional *SETDB1* deletion^36^, we observed that the strongest loss of H3K9me3 occurred at sites enriched for mHP1β in WT cells (Fig. 6c and Supplementary Fig. 10c). This supported our hypothesis that binding of mHP1β is preferentially targeted to genomic regions with high local concentration of H3K9me3.

### hHP1β dimerization maintains compacted chromatin states

To test whether dimerization is essential for the chromatin functions of HP1β, we first determined the subnuclear distribution of WT and mutant proteins in mouse fibroblasts. GFP-hHP1β largely localized to characteristic areas of pericentromeric heterochromatin enriched in H3K9me3. As deduced from our *in vitro* observations, the W42A, I161A or isolated CD mutant proteins, in contrast, showed diffuse distribution. However, heterologous dimerization of the NT–CD–HR via GST (NT, CD, HR(hHP1β)-GST) was sufficient to fully restore heterochromatin localization. In agreement with a contributing role of the HR, the NT, CD(hHP1β)-GST hybrid protein, did not completely copy the appearance of the WT factor (Supplementary Fig. 11a and b).

**Figure 7:**
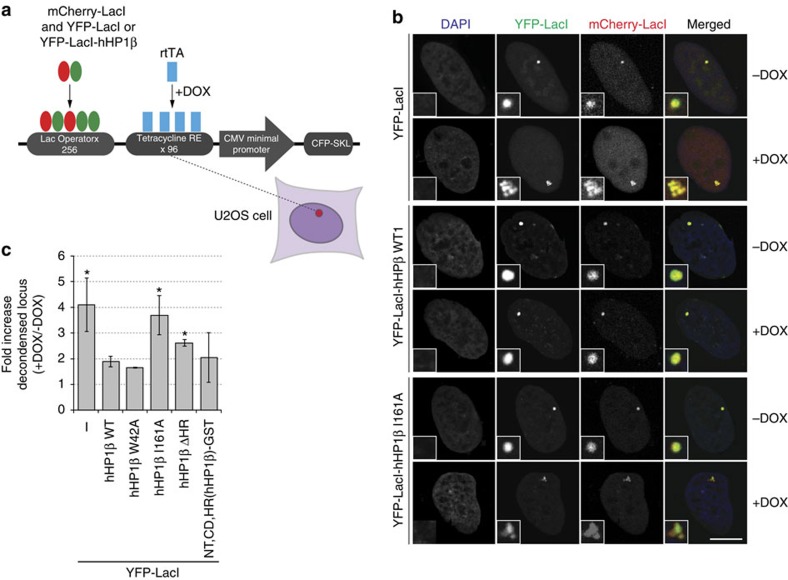
hHP1β dimerization is required for maintaining condensed cellular chromatin states. (**a**) Schematic diagram of the transgene system used to test chromatin compaction effects of hHP1β. A tandem array of the construct has been integrated into the genome of U2OS mammalian cells. Dox, doxycycline; rtTA, reverse tetracycline-controlled TET-VP16 transactivator, whose binding to tetracycline responsive elements (RE) is activated by doxycycline. (**b**) Together with mCherry-LacI, the indicated fusion proteins were transiently expressed in U2OS cells containing the chromatin reporter array as described in **a**. Representative confocal images in the absence or after induction of transcription from the array with doxycycline (Dox) are shown. DNA was stained with DAPI. Inlets show enlarged chromatin reporter array; scale bar, 10 μm. (**c**) Quantification of the results shown in **b** together with results of similar experiments using additional hHP1β mutant proteins. Averages of three independent experiments are plotted; error bars represent s.d.; *n*>300 for each condition; asterisks represent *P*<0.05 according to Student's *t*-test.

We then adopted a previously described ‘chromatin reporter system' that contains repetitive DNA arrays of LacO coupled to a regulatory transcription cassette (Fig. 7a). Integrated into the genome of U2OS cells these can be visualized after expression of mCherry-LacI as singular spot of heterochromatin^37^,^38^. Activation of transcription via doxycycline-induced expression of the rtTA activator resulted in decondensation of the array (Fig. 7b, YFP-LacI). Unfolding of the array on such activation was largely reduced by tethering of LacI-YFP-hHP1β WT but not the dimerization deficient I161A mutant to the locus (Fig. 7b,c). As expected, LacI-YFP-hHP1β W42A behaved very similar to the WT protein, as tethering of the exogenous protein to the locus was achieved by the LacI domain independently of CD/H3K9me3 interaction. Nevertheless, heterologous dimerization of the NT–CD–HR region of hHP1β via GST (NT, CD, HR(hHP1β)-GST) was sufficient to prevent unfolding of the array on activation. In agreement with contribution of the HR, the deletion of this domain resulted in an intermediate effect.

## Discussion

**Figure 8:**
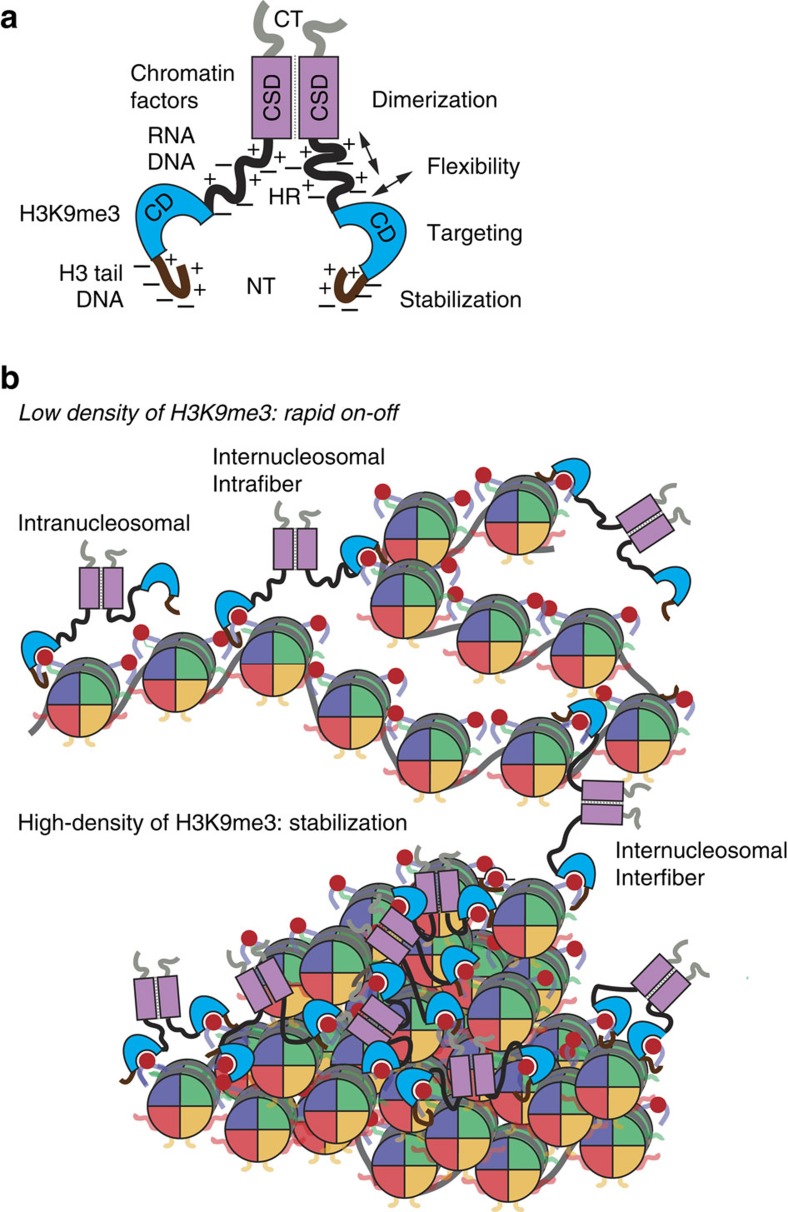
hHP1β is a paradigm HP1 protein. (**a**) Model summarizing the functional properties (right) and interactions of different domains of hHP1β. The balance of negatively and positively charged residues within the NT and HR is essential for specific H3K9me3–chromatin interaction. (**b**) Due to its flexible HR the CD of an hHP1β dimer can interact with the H3K9me3 marks of the same or different nucleosomes within the same or another chromatin fibre (top). The binding is stabilized by flexible and dynamic electrostatic interaction of the NT with the H3 tail. The high concentration of H3K9me3 in condensed and clustered oligonucleosomes (bottom) establishes multiple hHP1β binding possibilities. This steadies the interaction and stabilizes the compacted chromatin state.

We postulate that hHP1β exemplifies the fundamental interaction mode of HP1 proteins with chromatin. In our model (Fig. 8a,b) dimerization of the chromatin-binding module (that is, NT–CD) is required for increasing the avidity of the interaction by enabling longer residence time of HP1 on chromatin. While the CD is the main chromatin-binding domain, the charged NT provides additional anchoring via unspecific electrostatic interaction with the H3 tail. Flexibility within the HR allows binding of the chromatin-binding module to two H3 tails of the same nucleosome and different nucleosomes within the same or distinct chromatin fibres. Since neither the CD within a HP1 dimer nor the H3 tails are in a fixed orientation, the entropic effect of bivalent interaction onto binding strength is relatively mild (Table 1). Altogether these parameters result in highly dynamic and reversible interaction of HP1 with H3K9me3 chromatin that is in agreement with the structural parameters deduced for hHP1β binding to mononucleosomes^30^, recent single-molecule studies on hHP1α (ref. 14), as well as the highly mobile behaviour of HP1 proteins within heterochromatin of yeast and mammalian cells^39^,^40^,^41^.

In our model, modulation of the general chromatin-binding mode is brought about by varying sequences in the HR and NT domains as well as possibly their post-translational modification in other HP1 proteins (different variants, different species). We think that within these regions the overall charge but not a particular sequence is mainly dictating the chromatin-binding properties. Consistent with the requirement of a spacing and flexible linker (Fig. 8a), these domains are of relative low-sequence conservation and of varying length in different HP1 proteins (for example, HR(hHP1γ), 30 aa; HR(Swi6), 120 aa). In hHP1α the HR contains a surplus in positively charged residues (Supplementary Fig. 2a). Consequently, the protein shows limited discrimination between H3K9me0 and H3K9me3 chromatin due to CD-independent binding of DNA^12^. Phosphorylation of the NT neutralizes the surplus of positive charges and thereby directs an H3K9me3-specific chromatin-binding mode^13^. Interestingly, the NT and HR of hHP1α but not of hHP1β are extensively post-translationally modified^42^.

Swi6 contains an even more positively charged HR, which causes more pronounced binding to unmodified chromatin^29^, as well as an unusual long NT (Supplementary Fig. 2a). The superstoichometric recruitment of Swi6 to H3K9me3 chromatin observed by others^29^ and us has been attributed to ordered oligomerization of the protein via additional, induced CD–CD interaction^43^. However, we find that the Swi6(CD) is not sufficient for this effect (Fig. 5d,e). Contrariwise, our studies on hHP1β and work on hHP1α (ref. 14) clearly show that these proteins do not multimerize beyond dimerization in chromatin binding. Also, recent modelling approaches deduced an allosterically controlled mode of Swi6 in the absence of CD–CD interaction^44^. While the functional differences between Swi6 and mammalian HP1 proteins need to be further investigated, we note that this new interpretation of previous data is fully compatible with our general model.

How does the flexible and dynamic binding mediate the chromatin clustering effects of hHP1β We think the protein stabilizes condensed structures rather than inducing these *de novo*. It does not work as a locally directed and static chromatin clamp as suggested on the basis of the alleged binding mode of Swi6 (refs 45, 46). Yet, via a large number of transient and unordered bridging events HP1 keeps chromatin fibres associated in a plastic manner (Fig. 8b). First, local high concentration of H3K9me3, which is found in compacted chromatin regions, is required for stable hHP1α (ref. 14) and hHP1β (our work) chromatin association. Second, the main effect of HP1 dimerization and locally enriched H3K9me3 is in reducing the off-rate of the protein from chromatin (Fig. 2d, ref. 14). Third, HP1 is not able to induce chromatin clustering in absence of the H4 tail, which is important in higher order chromatin folding^23^. Bridging of H3 tails is either not possible in a relaxed chromatin state or is not sufficient for inducing chromatin compaction. We think that conformational fluctuations in chromatin fibres that allow transient local folding and unfolding are trapped by HP1 when yielding patches with high concentration of H3K9me3.

A major consequence of our interpretation is that cellular HP1-localization, -dynamics and -working mode are directed by the local density of H3K9me3, which might help explain the diverse biology ascribed to these proteins in hetero- and euchromatin^10^,^47^,^48^. For example, an intimate relationship exists between heterochromatin and the nuclear periphery in various metazoans^49^. H3K9 methylation appears to be the major driving force for the localization of genomic regions to the nuclear periphery and these generally overlap with lamina-associated domains^50^. Although a direct involvement in the recruitment to the nuclear periphery is controversial, the very dense chromatin areas might be clustered and stabilized by HP1. We think the dynamic mode of HP1 binding and actions on H3K9me3 chromatin allows flexible clustering and re-clustering of different regions of chromatin within the same or distinct chromosomes thereby contributing to the establishment and maintenance of heterochromatin. Importantly, such working mechanism is in agreement with an emerging view of high plasticity of nuclear chromatin in the absence of hierarchical organization^51^,^52^,^53^.

## Methods

### Plasmids

Plasmids containing DNA templates for chromatin reconstitution were a gift of Dr Daniela Rhodes (MRC Cambridge, UK). Plasmids for expression of recombinant histones from *Xenopus laevis* were a gift from Dr. Karolin Luger (University of Colorado, Boulder). Complementary DNAs (cDNAs) corresponding to hHP1β (GenBank NM_001127228) were amplified by PCR using a 5′ primer that introduces a His_6_ affinity tag and cloned into pET11a expression vector. Alternatively, the hHP1α(GenBank BC006821) and hHP1β cDNAs were cloned into pCold I (Takara Bio/Clontech, Saint-Germain-en-Laye, France). For point and deletion mutants, site-directed mutagenesis was carried out using the Stratagene QuickChange protocol or a Q5 site-directed mutagenesis kit (New England Biolab, Frankfurt, Germany) according to the manufacturers' instruction. cDNAs for other chimeric proteins were synthesized by Genewiz (South Plainfield, USA). YFP-LacI-NSL C1 and YFP-LacI C1 expression vectors were kind gifts from Dr Supriya Prasanth (University of Illinois, IL, USA)^38^. hHP1β WT, mutant and chimera cDNAs were amplified from the bacterial expression vectors by PCR and cloned into *Sal*I–*Bam*HI sites of the YFP-LacI C1 vector. A detailed list of the plasmids used in this study can be found in Supplementary Table 3.

### Western blotting

Anti-histone H3 (1/10,000; ab1791, Abcam, Cambridge, UK), swine anti-rabbit IgG (1/2500; P0399; DAKO, Hamburg, Germany), anti-His_6_-peroxidase (1/500; 1965085001; Roche, Mannheim, Germany); anti-hHP1β (1:2000; MAB3448, Merck Millipore, Schwalbach, Germany). Uncropped scans of gels and blots are provided in Supplementary Fig. 12.

### Expression and purification of recombinant HP1 proteins

For pET11a vectors, proteins were expressed using ZYM-5052 auto-inducing medium overnight at 30°C. For pColdI vectors, proteins were expressed in the same cells using standard LB medium overnight at 15°C in the presence of 0.4 mM IPTG. For production of pBpa-containing hHP1β, His-tagged hHP1β (N57X, Q158X) was expressed in the presence of pSUP-BPARS^54^ in *E. coli* BL21 DE3 in standard LB medium supplemented with 2 mM pBPA. Protein expression was induced at OD_600 nm_=1 by the addition of 1 mM IPTG for 4 h. All proteins were purified on Ni-NTA beads (Qiagen, Hilden, Germany) using standard protocols followed by anion exchange chromatography (MonoQ, GE Healthcare, Freiburg, Germany). Alternatively, proteins were purified using His-Pur Cobalt resin (Thermo Scientific, Braunschweig, Germany) with several rounds of high-salt washes (0.8 M NaCl) before elution. Purified proteins were dialyzed against PBS, 10% (v/v) glycerol, 2 mM DTT or a buffer containing 10 mM triethanolamine (pH 7.5), 150 mM NaCl, 1 mM DTT 30% (v/v) glycerol. Aliquots of purified protein were stored at −80 or −20°C, respectively.

### Expression and purification of recombinant histones

Core histones were expressed in *E. coli* BL21 (DE3) RIL cells using ZYM-5052 auto-inducing medium. Histone proteins were found exclusively in inclusion bodies, which were solubilized in unfolding buffer (7 M deionized urea, 20 mM Tris-HCl (pH 7.5), 10 mM DTT). The material was dialyzed against urea chromatography buffer (7 M deionized urea, 10 mM Tris-HCl (pH 7.5), 1 mM EDTA, 100 mM NaCl, 2 mM DTT, 0.2 mM PMSF) and loaded onto a Q Sepharose column in front of a SP Sepharose column (both GE Healthcare, Freiburg Germany). After washing with five column volumes of urea chromatography buffer, the Q Sepahraose column with bound DNA and contaminating proteins was removed. Histone proteins were eluted from the SP Sepharose column using a linear gradient from 0.1 to 0.6 M NaCl. Purified histones were dialyzed extensively against ddH_2_O, lyophilized and stored at −80°C.

### Native chemical ligation

0.2 mM of H3Δ1-20, A21C and 1 mM of N-terminal H3 peptide (1–20) with a C-terminal thioester group were ligated for 24 h in 100 mM potassium phosphate, 3 M guanidine-HCl, 0.5% (v/v) benzyl mercaptan, 0.5% (v/v) thiophenol, pH 7.9 at 25°C with vigorous mixing. The crude reaction mixture was dissolved into 25:75:0.1 acetonitrile/water/trifluoroacetic acid, diluted fivefold into SAU-200 buffer (7 M deionized urea, 20 mM sodium acetate (pH 5.2), 1 mM EDTA, 1 mM DTT, 200 mM NaCl), applied to a Hi-Trap SP-Sepharose high-performance cation exchange column (GE Healthcare, Freiburg, Germany), and eluted with a linear NaCl gradient from 200 to 600 mM. Protein samples were dialyzed extensively against 2 mM DTT at 4°C, lyophilized and stored at −80°C.

### Site-specific installation of H3K_C_9me3

H3K9C, C110A was expressed and purified as WT histones. 5 mM of mutant H3 was reduced for 1 h at 37°C in alkylation buffer (1 M HEPES, 4 M guanidium-HCl, 10 mM D/L-methionine, 20 mM DTT, pH 7.8). Alkylation reactions were performed at 50°C in the presence of 400 mM (2-bromoethyl)-trimethylammonium bromide (Sigma-Aldrich, Steinheim, Germany) in the dark with occasional mixing. After 2.5 h of incubation, 10 mM of fresh DTT was added and the reaction was allowed to proceed for another 2.5 h at 50°C. The alkylation reaction was quenched with 700 mM 2-mercaptoethanol, and the crude reaction mixture was diluted 50-fold into SAU-200 buffer. Alkylated histones were purified by anion exchange chromatography as described above.

### Reconstitution of histone octamers

Lyophilized purified WT core histones H2A, H2B, H4 and WT or modified H3 were dissolved in unfolding buffer and mixed to equimolar ratios. The histone mixture was extensively dialyzed at 4°C against RB high buffer (10 mM Tris-HCl, 1 mM EDTA, 2 M NaCl, 1 mM DTT, pH 7.5) with at least three changes of dialysis buffer. Histone octamers were concentrated to 10–20 mg ml^−1^ using Amicon Ultra centrifugal filter units (Millipore, Billerica, USA) and purified on a HiLoad 16/60 Superdex 200 prep grade gel filtration column (GE Healthcare, Freiburg, Germany). Peak fractions were pooled and concentrated to at least 2 mg ml^−1^. Histone octamers were stored in 50% (v/v) glycerol at −20°C.

### Reconstitution of chromatin templates

Mononucleosomes were reconstituted on a 187 bp DNA fragment containing the ‘601' sequence flanked on each site by a linker of 20 bp^55^. Oligonucleosomes were reconstituted on a 12 × 200 bp × 601 template^56^. Plasmids carrying the respective inserts were purified using a Giga kit (Qiagen, Hilden, Germany). Templates for reconstitution were released from plasmids by restriction digest with *Bso*BI for mononucleosomes and a mix of *Dde*I, *Bfu*CI, *Hae*II and *Eco*RI for oligonucleosomes. To separate vector backbone from DNA used for chromatin assembly stepwise precipitation with polyethylene glycol (PEG) 6000/0.5 M NaCl was carried out (final PEG concentration 2–9% and 20% (w/v)). DNA pellets were washed with 70% (v/v) ethanol and dissolved in water. Double stranded, short, biotinylated DNA linkers were ligated to the DNA template before chromatin reconstitution.

Histone octamers were dialyzed for at least 3 h against RB high buffer. DNA templates were added in a molar ratio of 0.8–1.2 (concentration of nucleosome positioning sites to histone octamers). The reaction mixtures were dialyzed against RB high buffer that was continuously replaced by RB low buffer (10 mM Tris-HCl, 1 mM EDTA, 10 mM NaCl, 1 mM DTT, pH 7.5) over a 36 h period using a peristaltic pump. Quality and nucleosome saturation of reconstitution reactions were monitored by agarose gel electrophoresis and analytical ultracentrifugation. Reconstituted chromatin templates were extensively dialyzed against TEAE buffer (10 mM triethanolamine HCl pH 7.5, 0.1 mM EDTA, pH 7.5) and stored at 4°C.

### Electrophoretic mobility shift assay

50 ng 150 bp DNA amplified from the backbone of pUC18 was incubated with 0, 3, 9 μM recombinant protein in 10 μl EMSA buffer (10 mM Tris-HCl (pH 8.0), 100 mM NaCl, 5 mM MgCl_2_) for 20 min at room temperature (RT). 2 μl 50% (v/v) glycerol was added and samples were loaded onto 6% native PAGE gels. After electrophoresis, DNA was visualized by staining with 1 × SYBR Gold/0.25 × TBE for 20 min at RT. Images were captured using a ChemiDoc MP Imaging System (Bio-Rad, München, Germany).

### Chromatin coprecipitation

6.7 nM oligonucleosomes were incubated with 1 μM recombinant protein in 100 μl binding buffer (10 mM triethanolamine (pH 7.5), 150 mM NaCl, 5 mM MgCl_2_, 0.1 mM EDTA, 0.1% (v/v) Triton-X100) for 1 h on ice. Precipitated chromatin complexes were recovered by centrifugation at 16,100*g* for 30 min at 4°C. Pellets were washed once with 0.5 ml binding buffer and centrifuged for 15 min at 4 **°**C. Precipitated material was resuspended in 10 μl 1 × SDS loading buffer and boiled for 5 min before running on SDS–PAGE.

### Chromatin sedimentation

For saturating conditions, 2.7 nM oligonucleosomes were incubated with 5 μM hHP1β proteins in 100 μl sedimentation buffer (10 mM triethanolamine (pH 7.5), 150 mM NaCl, 0.1 mM EDTA) for 1 h on ice. For non-saturating conditions, 3 μM recombinant proteins were used and the NaCl concentration in the sedimentation buffer was lowered to 100 mM. After incubation, 40 μl per sample was placed into a well of a 96-well plate and kept as ‘input'. The rest was centrifuged at 16,100*g* for 30 min at 4°C. 40 μl supernatant was carefully removed and placed into a 96-well plate (‘output'). For quantification of chromatin DNA, the input and output samples were incubated with 40 μl 0.5 μg ml^−1^ ethidium bromide and fluorescence was measured on a Plate CHAMELEON II fluorescence plate reader (Hidex, Turku, Finland) using a 360 nm (±5 nm) excitation filter and a 612 nm (±5 nm) emission filter. For each sample the ratio of DNA concentrations after and before centrifugation (output/input) was calculated.

### Pull-down experiments

4 μg C-terminally biotinylated histone H3 peptides (aa 1–20) or 1 μg of biotinylated nucleosomes or oligonucleosomal arrays were incubated with 40 μl Streptavidin MagnaSphere Paramagmetic beads (Promega, Mannheim, Germany) in 350 μl binding buffer (10 mM triethanolamine (pH 7.5), 150 mM NaCl, 0.1 mM EDTA, 0.1% (v/v) Triton-X100) supplemented with 1 mg ml^−1^ BSA for 2.5 h at RT. Unbound material was removed by three washes with 0.5 ml binding buffer. Charged beads were then incubated with 100 pmol recombinant protein in 350 μl binding buffer for 1 h at 4°C on a rotator. Beads were washed three times with 0.5 ml binding buffer for 3 min per wash at 4°C. Bound proteins were eluted in 20 μl 1 × SDS loading buffer and by boiling for 5 min.

### Fluorescence correlation spectroscopy

All fluorescence measurements were performed with a pulsed custom-built mode-locked Ti:Sa-Laser and a confocal two-photon fluorescence microscope setup. Detailed description of the experimental setup and the measurement procedure are available on request. In all cases 10 nM of the acceptor labelled species, ATTO 610-labelled nucleosomal array, was incubated with 20–200 μM of recombinant hHP1β in 10 μl of 10 mM triethanolamine HCl (pH 7.5), 0.1 mM EDTA, 150 mM NaCl at 4°C. For each sample the fluorescence correlation curve, fluorescence intensity and anisotropy values were determined at 22°C. Every data point was averaged over a minimum of 15 single measurements of 10 s each. Excitation was by two-photon mode.

The fluorescence data were analysed using the correlation function 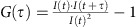, and the equation for free diffusion of particles 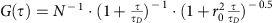, where I(*t*) is the fluorescence intensity measured at time *t*, *N* the particle number, *τ*_*D*_ the diffusion time, and *r*_0_=0.25 the axis ratio of the focal region, considering the dimensions of a Gaussian beam waist.

### Fluorescence polarization measurements

Experiments were performed in 10 mM triethanolamine HCl (pH 7.4), 150 mM NaCl, 0.1 mM EDTA, 2 mM DTT. Titration series of 10 μl volume in 384-well plates were read multiple times on a Plate CHAMELEON II plate reader (HIDEX Oy, Turku, Finland). Multiple readings and independent titration series were averaged after data normalization^57^.

### Isothermal calorimetry

ITC measurements were performed on a iTC200 calorimeter (Microcal, Malvern, UK) at 25°C in binding buffer (10 mM triethanolamine (pH 7.5), 20 or 150 mM NaCl, 0.1 mM EDTA). Reaction heats were recorded by sequences of 37 injections of 1.8 mM hHP1β, spaced at 120 s intervals, into 250 μl of 60 μM H3 peptides, nucleosomes or oligonucleosomal arrays under constant stirring at 1,000 r.p.m. (injection #1–10: 0.5 μl each; injection #11–32: 1 μl each; injection #33–37: 2 μl each). Heats of dilution, obtained by the titration of hHP1β into buffer, were substracted from raw data before analysis. Raw data were integrated, normalized and the apparent heat change (Δ*q*) of the reaction was plotted against the molecular ratio using the Origin software. For the determination of apparent enthalpy changes (ΔH_app_), the molar association constant (*K*_*A*_), and the stoichiometry (*n*) of the interaction, non-linear least-square fitting of the Δ*q* values was performed by the Origin software using a binding model of one set of identical binding sites.

### Surface plasmon resonance

SPR measurements were performed on a Biacore 2000 instrument (GE Healthcare, Buckinghamshire, UK) at 298 K in 10 mM triethanolamine (pH 7.5), 150 mM NaCl, 0.1 mM EDTA, 1 mM DTT, 0.005% (v/v) Tween-20. Biotinylated ligands were immobilized on streptavidin-coated sensor chips SA (GE Healthcare, Buckinghamshire, UK). Before immobilization, sensor chips were conditioned as described by the manufacturer. To obtain low surface densities (5–24 RU), 5 nM biotinylated H3 peptide was injected at a low rate of 10 μl min^−1^ using varying contact times. High surface densities (>700 RU) were prepared by injecting 3.5 mM peptide (10 μl min^−1^) until the SA surface was saturated. Peptide-containing chips were regenerated by applying three short pulses of 0.05% (w/v) SDS followed by an injection of 1.5 M NaCl for baseline stabilization. For binding assays, hHP1β proteins were injected at 24–50 μM (serial twofold dilutions) for 2 min (30 μl/min). Dissociation was recorded for up to 5 min. A streptavidin surface without ligands served as reference. In addition, blank runs were performed for double referencing. Data evaluation was performed by steady-state analysis assuming a Langmuir 1:1 binding model using BIAevaluation 4.1 and Prism 5.04 (GraphPad, CA, USA) software. Binding to the unmodified peptides was analysed with maximum binding (RU_max_) set to the corresponding values obtained with the modified peptides.

### Scanning force microscopy

Nucleosomal arrays and recombinant hHP1β were dialyzed against SFM buffer (5 mM triethanolamine (pH7.5), 50 mM NaCl, 0.1 mM EDTA). The recombinant proteins at different concentrations (between 0 and 5 μM) were preincubated with oligonucleosomes at a DNA concentration of 50 ng ml^−1^ in SFM buffer for 1 h at 4°C, and the samples were fixed with 0.05% (v/v) fresh glutaraldehyde (Electron Microscopy Sciences, PA, USA) at 4°C overnight. Fixed samples were extensively dialyzed against TEAE buffer (5 mM triethanolamine (pH 7.5), 0.1 mM EDTA). 10 μl of dialyzed sample was deposited on *ca.* 1 cm^2^ of freshly cleaved mica (Plano). After 5 min incubation at RT, the mica was washed with 200 μl of water (high-performance liquid chromatography grade) and air-dried. Images were recorded in air on a Nanoscope V Multi Mode scanning force microscope (Veeco, NY, USA) using an ‘E'-scanner with maximum scan size of 15 × 15 μm and silicon-etched probe tips with a typical spring constant of 40 N m^−1^ and a typical resonance frequency of 325 kHz (NSC15, MikroMasch) in tapping mode.

### Chemical cross-linking and mass spectrometry

13.4 nM H3K_C_9me3 oligonucleosomes were mixed with 2 μM hHP1β in 1 ml cross-linking buffer (20 mM sodium phosphate (pH 6.4), 100 mM NaCl, 0.1% (v/v) Triton-X100) and incubated for 30 min at RT. Freshly prepared EDC (Sigma-Aldrich, Steinheim, Germany) was added to 15 mM and incubated for 30 min at RT. In the case of a higher chromatin: hHP1β ratio, 15 μM of hHP1β and 35 mM EDC were used. MgCl_2_ was then added to 5 mM and chromatin and crosslinked/bound hHP1β were recovered by centrifugation at 16,100*g* for 30 min at 4°C. The pellet was washed once with the EDC buffer containing 5 mM MgCl_2_ and dissolved in 20 mM Tris-HCl (pH 8.0), 0.8% (w/v) SDS. The sample was denatured for 15 min at 70°C, then treated with 20 mM DTT (Merck, Mannheim, Germany) for 30 min at 56°C, which was followed by treatment with 20 mM iodoacetamide for 20 min at RT (Sigma-Aldrich, Steinheim, Germany). The SDS concentration of the sample was adjusted to ≤0.1% (w/v) and 3 μg trypsin (Promega, Mannheim, Germany) was added for 16 h at 37°C. Tryptic peptides were purified using Sep-Pak SPE C18 cartages (Waters, Dublin, Ireland) and separated using size-exclusion chromatography^58^,^59^. Each peptide-containing fraction was analysed on an LTQ-Orbitrap Velos mass spectrometer (Thermo Fisher Scientific, Bremen, Germany)^60^. Data analysis was performed using pLink^61^. All cross-link-containing peptide fragment spectra were manually evaluated^62^. Crosslinks were visualized with xiNET (http://crosslinkviewer.org).

### Ultraviolet-mediated protein–protein cross-linking

54 nM oligonucleosomal arrays were incubated with 8 μM standard or photo crosslinkable hHP1β in 100 μl binding buffer (10 mM triethanolamine (pH 7.5), 150 mM NaCl, 0.1 mM EDTA, 0.1% (v/v) Triton-X100) for 30 min on ice. The mixtures were placed into wells of a custom-made metal sample holder on ice and irradiated with ultraviolet light (365 nm, 8 W lamps (Vilber Lourmat, Eberhardzell, Germany); the distance between the light source and sample was set ∼5 cm) for 3 × 10 min with intermittent mixing. After irradiation, the samples were collected into 1.5 ml low-binding tubes (Eppendorf, Hamburg, Germany). MgCl_2_ was added to 5 mM and chromatin and bound proteins were recovered by centrifugation at 16,100*g* for 30 min at 4°C. Pellets were washed once with 0.5 ml binding buffer and resuspended in 10 μl 1 × SDS loading buffer and boiled for 5 min.

### Generation of HP1β–EGFP mESCs

The mouse HP1β coding sequence was cloned into the pCAGGS–EGFP–IRES–Puro plasmid containing a chicken beta-actin promoter. Stable mHP1β-GFP ESC lines were generated by transfecting 6 μg *Pvu*I-linearized vector into mouse WT ESCs.

### HP1β ChIP-seq

For crosslink MNase ChIP of HP1β, 10^7^ TT2 WT mouse ES cells were collected. Cells were crosslinked with 1% (v/v) formaldehyde in 10 ml of PBS for 10 min at RT, then quenched with 0.125 M glycine for 5 min. Cells were washed 1 × with PBS, and resuspended in 1 ml of EZ Nuclei Isolation Buffer (Sigma-Aldrich, Steinheim, Germany). The cytoplasmic supernatant was discarded and the pellet was flash frozen with liquid N_2_. Nuclei were washed 1 × with MNase wash buffer (50 mM Tris-HCl (pH 8.0), 1.5 mM DTT, 1 mM PMSF, 1 × Protease Inhibitor Cocktail (Roche, Indianapolis, USA)) and resuspended in MNase Digestion Buffer (10 mM Tris-HCl (pH 7.5), 4 mM MgCl_2_, 1 mM CaCl_2_, 1 mM PMSF, 1 × Protease Inhibitor Cocktail). Chromatin was digested to mono-, di- and tri-nucleosomes with the addition of micrococcal nuclease (NEB, Ipswich, USA) and incubated at 37°C for 7 min. Digestion was quenched with the addition of EDTA to a final concentration of 10 mM. Cells were then lysed with 1 ml of IP Buffer (0.5% (v/v) NP-40, 0.1% (v/v) Sodiumdeoxycholate, 0.1% (v/v) SDS, 150 mM NaCl, 10 mM EDTA, 1 mM PMSF, 1 × protease inhibitor cocktail) at 4°C on a rotator for 1 h. Cell debris was then pelleted, and the soluble chromatin fraction was precleared with Protein A/G Dynabeads (Life Technologies, Carlsbad, USA) at 4°C, rotating for 2 h. During preclearing, antibody–bead complexes were prepared using antibodies specific for HP1β (clone D2F2, NEB #8676) and Protein A/G Dynabeads each, in IP buffer at 4°C, rotating for 2 h. 10^6^ cell equivalent of chromatin was added to each antibody–bead complex, rotating overnight. Beads were washed and eluted in the presence of RNase A. To reverse cross-linking, eluted DNA was incubated with Proteinase K and high salt O/N at 65°C. DNA was then purified with phenol:choloroform.

A third of ChIP and input DNA (10–20 ng total) underwent end repair with T4 and Klenow, A-tailing and adaptor ligation. Following ligation, libraries were amplified using primers to the adaptors with eight PCR cycles. Resulting PCR was then purified on 2% EX Gel (Life Technologies, Carlsbad, USA) at the 200–700 bp range, and quantified on Qubit and Agilent Tapestation.

For ChIP from GFP-HP1β mESC fixation was done using double cross-linking with Di(N-succinimidyl)-glutarate (2 mM final concentration) and 1% (v/v) formaldehyde^63^. An α-EGFP antibody (Invitrogen, A11122, 5 μg per IP) was used for IP. 10 ng DNA from ChIP sample was used for library preparation. Briefly, end repair, dA tailing and adapter ligation were performed following the NEBNext ChIP-seq Sample Prep Master Mix (New England Biolabs, Ipswich, USA) guidelines and enriched by PCR amplification (18 cycles) before sequencing. 100 bp paired-end sequencing was performed on Illumina Hi-Seq 2000.

### Data sets and read processing

Following published data sets were downloaded from NCBI/GEO for this study: SPR899601/ H3K9me3 SETDB1^f/−^ and SPR899602/H3K9me3 SETDB1^−/−^ both done with anti-H3K9me3 antibody (ab8898)^36^. Reads were processed and aligned to the mouse reference genome (mm9) using QuasR in R. Genomic alignment was performed twice using BOWTIE with the following parameters: --strata, --best and -m 1 or -m 99 to account for reads that map uniquely or up to 99 times in the mouse genome, respectively^64^. For genome-wide comparisons, only uniquely mapping reads were used. Reads mapping up to 99 times were used for ERV analysis and genome browser visualization.

### Genome-wide analysis

The mouse genome was partitioned in 1 kb-sized windows, overlapping by 500 bp. The number of mapped reads was counted for each window. Log_2_-fold enrichments were calculated as following log_2_ (N_reads_IP+psc) – log_2_ (N_reads_input+psc). *psc* is a pseudocount constant of 8 to remove low-coverage noise. To exclude potential biases due to annotation issues, the following genomic windows were discarded from this analysis: windows overlapping with satellite repeats and windows not sufficiently covered in input samples. Finally, 3.46 × 10^6^ 1 kb-sized windows were used.

ERV-type repeat regions were obtained from www.repeatmasker.org. Cumulative read counts overlapping with ERV elements were calculated per repeat instance. Readcounts were normalized to instance length. Log_2_-fold enrichments were calculated as above. Only instances that contained more than one read in all experiments were used.

### Transfection of cells and fluorescence microscopy

U2OS 2-6-3 CLTon cells^38^ were kindly provided by Dr Supriya Prasanth (University of Illinois, IL, USA) and maintained in DMEM (high glucose+GlutaMax) (GIBCO/Invitrogen, Darmstadt, Germany) supplemented with 10% (v/v) TET system approved fetal bovine serum (Clontech, Saint-Germain-en-Laye, France), 50 μg ml^−1^ hygromycin (Carl Roth, Karlsruhe, Germany) and 200 μg ml^−1^ G418 (Clontech, Saint-Germain-en-Laye, France). For fluorescence microscopy, cells were grown on coverslips in a six-well plate without antibiotics. 500 ng plasmid DNA was transfected using Lipofectamine LTX and Plus reagent (Invitrogen, Darmstadt, Germany). 20 h post transfection, cells were treated with 1 μg ml^−1^ doxycycline for 24 h for induction of rtTA expression. Cells were washed twice with PBS and fixed with 3.7% (v/v) formaldehyde/PBS for 10 min at RT. Cells were permeabilized with 0.5% (v/v) Triton-X100 (PBS) for 5 min at RT and incubated with 200 ng ml^−1^ DAPI (PBS) for 5–10 min. Cells were mounted in Vectashield (Vectorlabs, CA, USA). Images were acquired using a Leica SP5 confocal microscope with a 63 × oil immersion lens (Leica Mikrosysteme, Wetzlar, Germany). *P* values (according to Student's *t*-test) were calculated using GraphPad QuickCals (GraphPad, CA, USA).

## 

## Additional information

**Accession codes**: HP1β ChIP-seq datasets were deposited at the Gene Expression Omnibus (GEO) of National Center for Biotechnology Information under the accession number GSE71114.

**How to cite this article:** Hiragami-Hamada, K. *et al*. Dynamic and flexible H3K9me3 bridging via HP1β dimerization establishes a plastic state of condensed chromatin. *Nat. Commun.* 7:11310 doi: 10.1038/ncomms11310 (2016).

## Supplementary Material

Supplementary Information Supplementary Figures 1-12, Supplementary Tables 1-3, Supplementary Note 1, Supplementary Methods and Supplementary References

Supplementary Data 1 List of pairs of chemically crosslinked residues of hHP1β and core histones within H3 K9me3 oligonucleosomes using 1-Ethyl-3-[dimethylaminopropyl] carbodimine (EDC) identified by mass spectrometry.

## Figures and Tables

**Table 1:** Interaction parameters of hHP1β WT and mutant proteins with the H3K9me3-modification in different biochemical environment as deduced by different methods.

			ITC				SPR
			20 mM NaCl		150 mM NaCl		150 mM NaCl
			*K*_d_ (μM)	*N*	*K*_d_ (μM)	*N*	*K*_d_ (μM)
Peptide	H3K9me3	WT	1.4±0.6	0.9±0.0	3.3±0.2	1.0±0.0	0.7±0.1*
		I161A	1.1±0.5	1.0±0.1	3.4±0.2	0.9±0.0	7.0±0.4
		CD	—	—	3.3±0.5	1.1±0.1	6.3±0.9
Peptide	H3K_C_9me3	WT	11±4	1.0±0.0	21±3	1.0±0.1	—
		I161A	—	—	—	—	—
Mononucleosome	H3K9me3	WT	—	—	—	—	1.0±0.2*
		I161A	—	—	—	—	5.2±0.6
Mononucleosome	H3K_C_9me3	WT	59±8	2.1±0.3	22±8	1.4±0.3	—
		I161A	223±30	1.1±0.4	58±17	1.2±0.3	—
Oligonucleosome	H3K_C_9me3	WT	NB		11±2	1.9±0.1	—
		I161A	NB		132±24	1.1±0.3	—

NB, not binding; ITC, isothermal titration calorimetry; N, stoichometry of interaction; Kd, apparent dissociation constant; SPR, surface plasmon resonance; WT, wild type.

^*^Based on fitting of titration data to a one site-specific binding model (Supplementary Note 1).
