# Supplementary material for: Dynamic and flexible H3K9me3 bridging via HP1β dimerization establishes a plastic state of condensed chromatin
Source: Nat Commun. 2016 Apr 19;7:11310. doi: 10.1038/ncomms11310 (PMC4838890; doi:10.1038/ncomms11310)
Supplement: Supplementary Information — Supplementary Figures 1-12, Supplementary Tables 1-3, Supplementary Note 1, Supplementary Methods and Supplementary References [file ncomms11310-s1.pdf]

SUPPLEMENTARY FIGURES

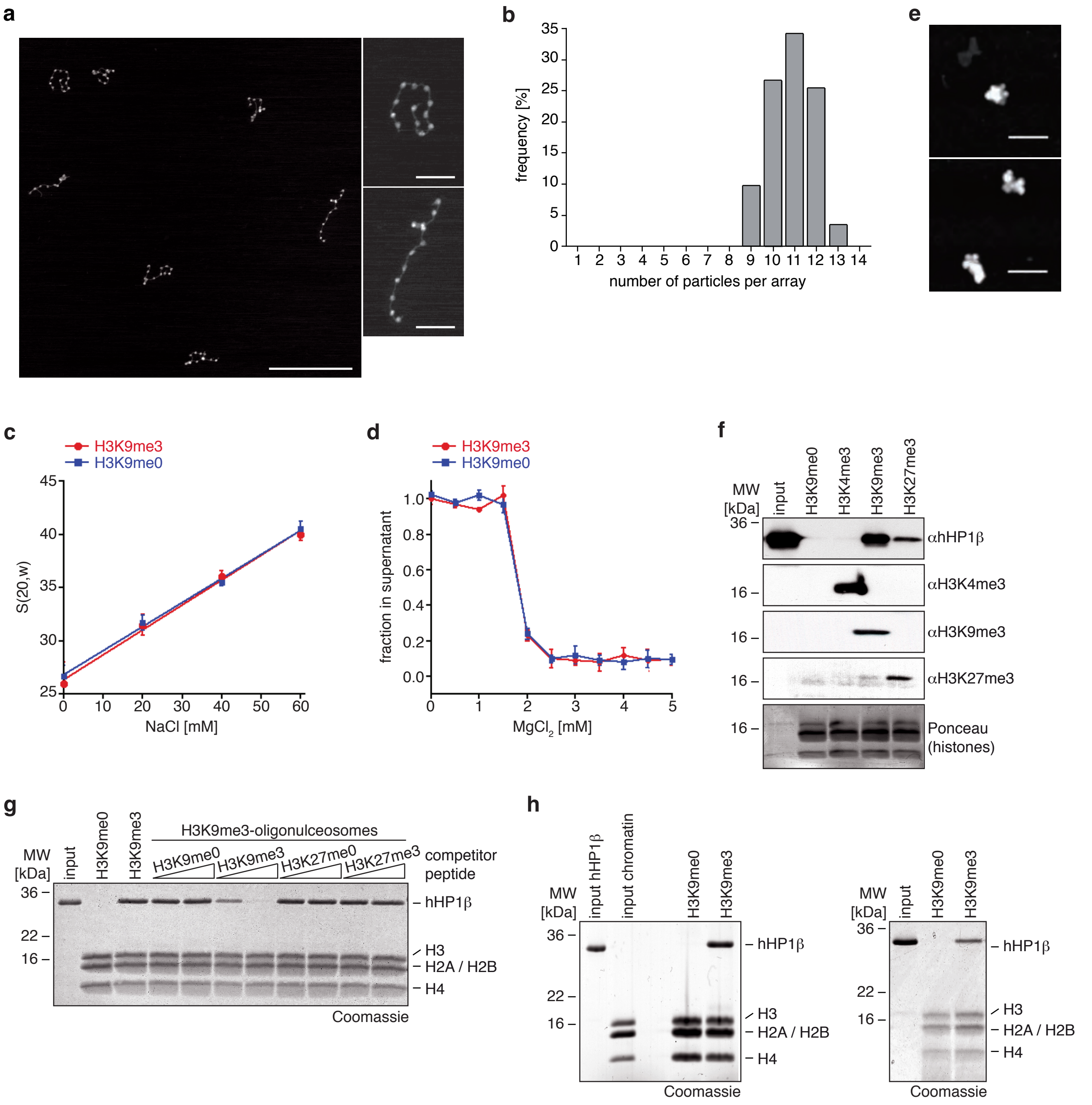

Supplementary Figure 1: Characterization of recombinant chromatin arrays and their interaction with hHP1β.

(**a**) Scanning force microscopy images of recombinant chromatin reconstituted at a 1.1 : 1.0 octamer : DNA ratio in presence of 50 mM NaCl and fixed with 0.05 % (v/v) glutaraldehyde. Field shot (scale bar corresponds to 500 nm) and two selected individual arrays (scale bar corresponds to100 nm) are shown.

(**b**) Distribution profile of the number of nucleosomes visible on reconstituted arrays as analyzed by scanning force microscopy. n > 100.

(**c**) Condensation behavior of H3K9me0- and H3K9me3-oligonucleosomes at different concentration of NaCl was analyzed by analytical ultracentrifugation. Corrected sedimentation coefficients obtained by SEDFIT analysis are plotted; error bars represent s.d.; n = 3.

(**d**) Analysis of $MgCl_2$-dependent chromatin interfiber clustering. DNA remaining in the supernatant after centrifugation was incubated with EtBr and measured by fluorescence reading. Data were normalized to amounts present in the input. Averages of three independent experiments are plotted; error bars represent

s.d.; n = 3.

(**e**) H3K9me0 (left) and H3K9me3 (right) oligonucleosomes at 150 mM were fixed with 0.05 % (v/v) glutaraldehyde, spotted on mica surfaces and analyzed by scanning force microscopy. Scale bar corresponds to 100 nm.

(**f**) Coprecipitation of hHP1β WT with oligonucleosomes of the indicated modification status. Precipitated material was analyzed by western blotting. Ponceau staining of the region of the western blot membrane containing histones is shown as loading control.

(**g**) Coprecipitation of hHP1β WT with H3K9me3-oligonucleosomes in presence of H3-tail peptides of the indicated modification status at 2 : 1 and 4 : 1 molar excess over H3K9me3-marks of the chromatin template. Precipitated material was run on SDS-PAGE and stained with Coomassie Blue.

(**h**) Coprecipitation of hHP1β WT with oligonucleosomal arrays reconstituted on different DNA templates. Right, chromatin on linearized pUC18 plasmid; left, chromatin on 12 x 5S rDNA. Reactions were separated by SDS-PAGE and stained with Coomassie Blue.

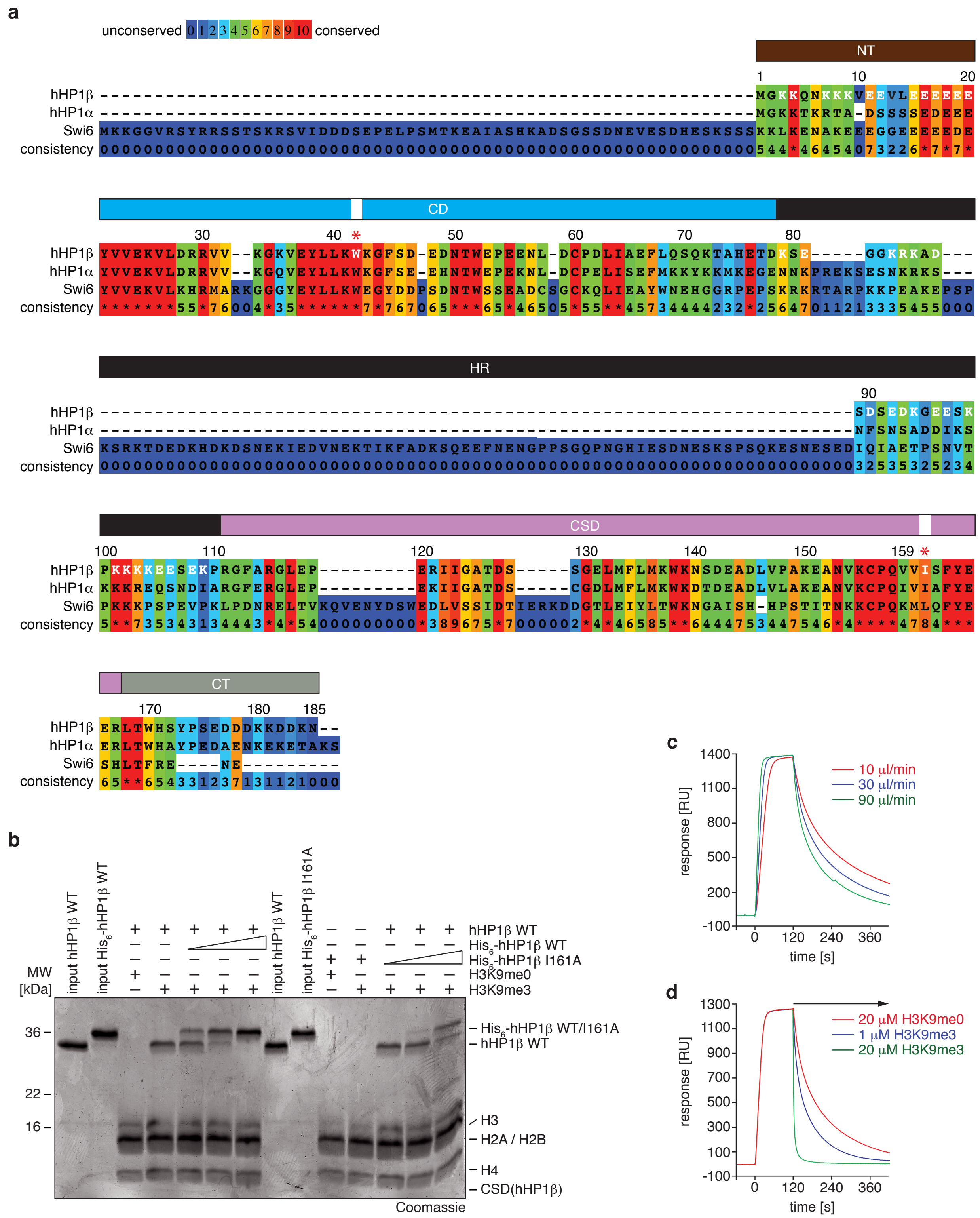

Supplementary Figure 2: Bivalent interaction of hHP1β with H3K9me3-chromatin.

(**a**) Sequence alignment of hHP1β, hHP1α and *S. pombe* Swi6 using PSI-BLAST. Boundaries of domains are reflected by the scheme on the top (see also **Figure 1**). Numbering of amino acid positions is according to hHP1β. Residues of hHP1β mutated in this study are highlighted (white); positions of important mutations in the CD (W42, abolishing H3K9me3 binding) and CSD (I161A, abolishing dimerization) are indicated (red asterisks in alignment and white boxes in scheme).

(**b**) Competitive binding of hHP1β WT and I161A to H3K9me3-oligonucleosomes was analyzed by

chromatin coprecipitation. 0.5 μM untagged hHP1β WT or I161A was pre-incubated with oligonucleosomal arrays of the indicated modification status (concentration of nucleosomes 0.8 μM). After 30 min His_6-tagged hHP1β WT or I161A were added to 0.5 μM, 5 μM, or 50 μM for 30 min. After addition of 5 mM $MgCl_2$ precipitates were recovered by centrifugation and separated by SDS PAGE. Gel was stained with Coomassie Blue.
(**c**) 0.1 μM hHP1β WT was analyzed on an SPR surface with high density of H3K9me3-peptide (950 RU) at different flow rates.
(**d**) 0.1 μM hHP1β WT bound to an SPR surface with high density of H3K9me3-peptide (950 RU) was washed off (indicated by the arrow) by injection of different free H3-peptides.

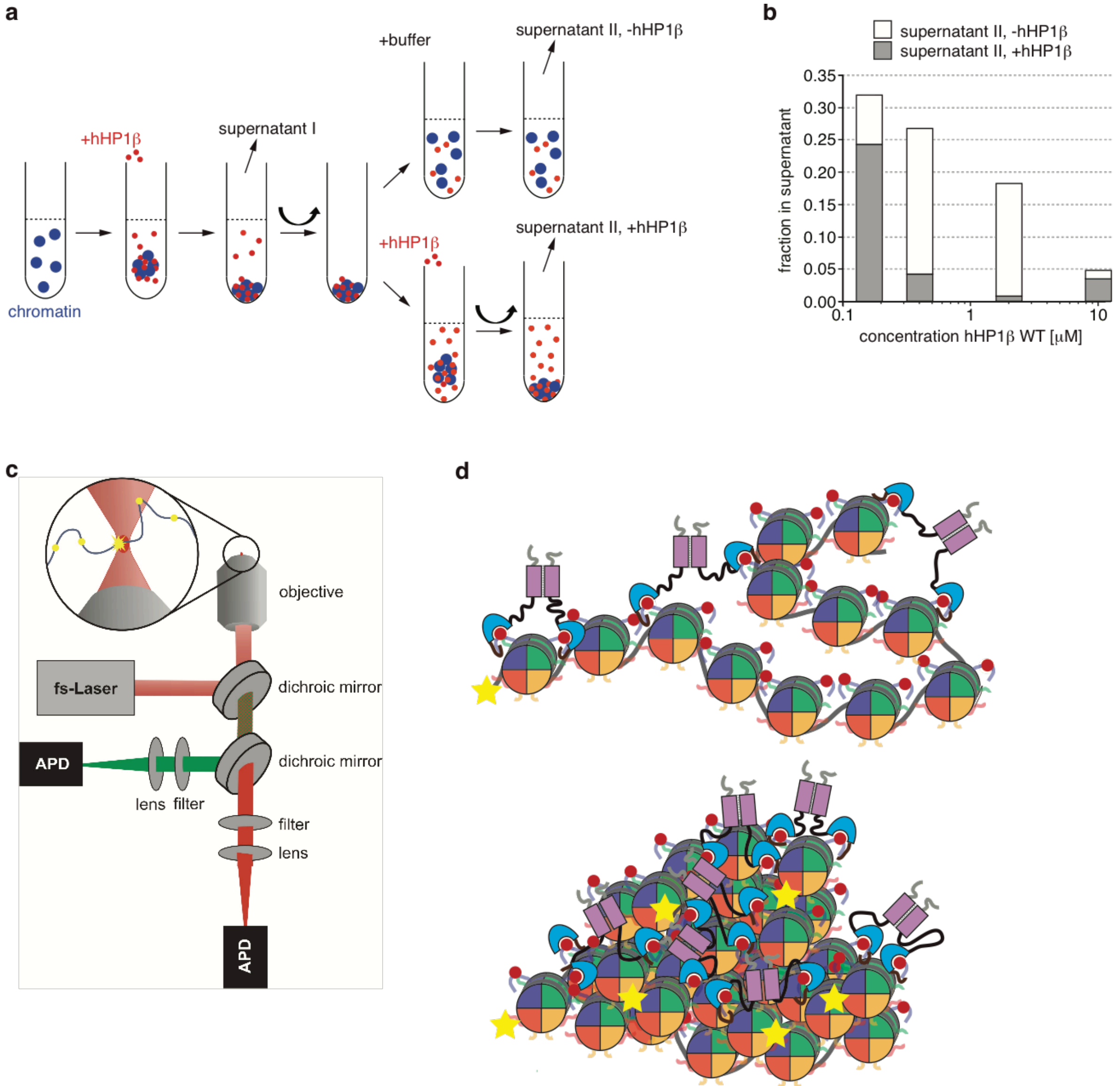

Supplementary Figure 3: Analysis of chromatin clustering mediated by hHP1β.
(**a**) Schematic representation of a stepwise precipitation and resolubilization experiment.
(**b**) Results of stepwise precipitation and resolubilization after clustering H3K9me3-oligonucleosomes by hHP1β at different concentrations. DNA remaining in the supernatant after centrifugation was incubated with EtBr and measured by fluorescence reading. Data were normalized to amounts present in the input. Results of a representative experiment are shown.
(**c**) Instrumental setup used for two-photon fluorescence correlation spectroscopy (FCS). The beam of a pulsed Ti:Sa-Laser is reflected into a water immersion objective (O) via a dichroic mirror (M). The fluorescence light transmitted through this dichroic mirror is split up by a second dichroic mirror. Blow-up shows the trajectory of a diffusing molecule. F: filter, L: lens, M: dichroic mirror, O:objective, PD: photo detector.
(**d**) Scheme of the strategy used for FCS measurements. DNA of oligonucleosomes was labeled at the 5'-end with ATTO 610.

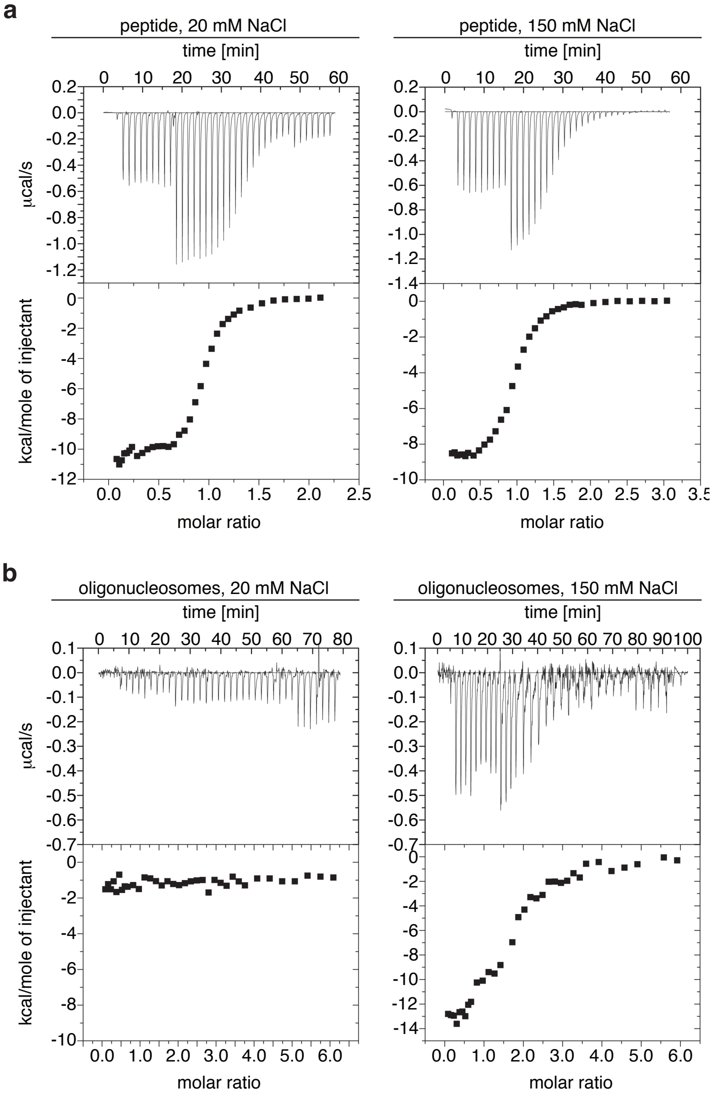

Supplementary Figure 4: Quantitative analysis of interaction of hHP1β with H3K9me3-peptides and oligonucleosomes.

(**a**) ITC measurements of H3K9me3-peptide/hHP1β WT interaction at 20 mM NaCl (left) and 150 mM NaCl (right). Upper panels, raw data of heat release upon stepwise injection of recombinant protein into solution of peptide; lower panels, plots of integrated heats of protein injections vs. molar ratio of hHP1β WT per peptide calculated by MicroCal software. Injections #1-10: 0.5 µl; injections #11-32: 1 µl; injections #33-37: 2 µl.

(**b**) ITC measurements as in (a) but with $H3K_C9me3$-oligonucleosomal arrays.

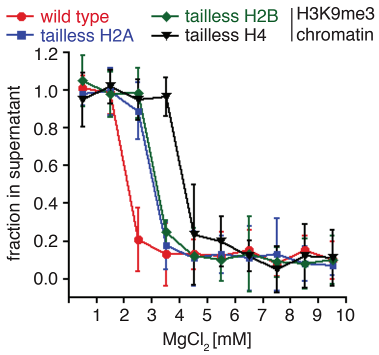

Supplementary Figure 5: Characterization of oligonucleosomal arrays containing tailless histones. Analysis of $MgCl_2$-dependent chromatin interfiber clustering of oligonucleosomal arrays containing H3K9me3 and wild type or tailless core histones. DNA remaining in the supernatant after centrifugation was incubated with EtBr and measured by fluorescence reading. Data were normalized to amounts present in the input. Averages of three independent experiments are plotted; error bars represent s.d.; n = 3.

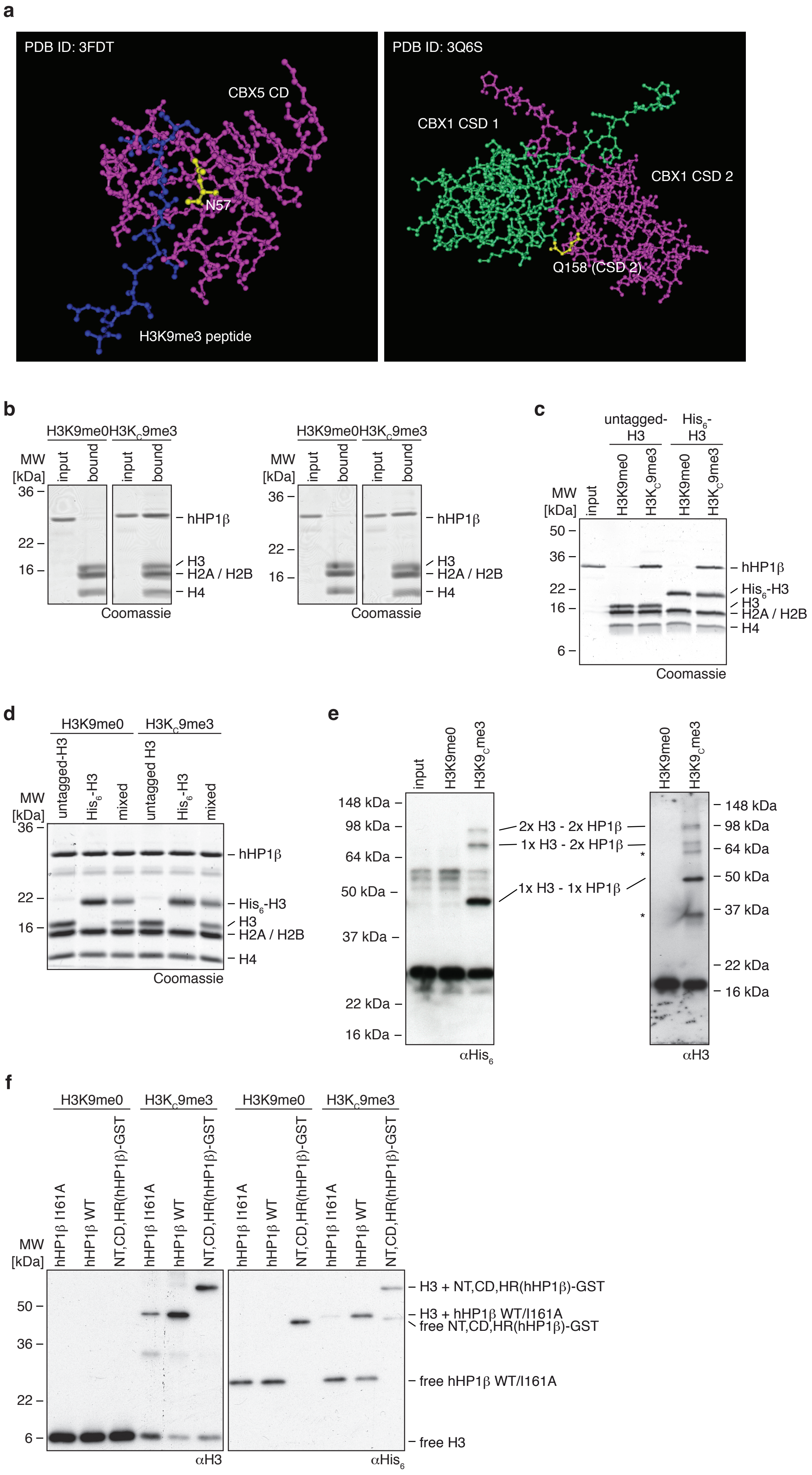
a
PDB ID: 3FDT
CBX5 CD
N57
H3K9me3 peptide
PDB ID: 3Q6S
CBX1 CSD 1
CBX1 CSD 2
Q158 (CSD 2)
b
H3K9me0
H3K$_C$9me3
MW [kDa]
input
bound
hHP1β
H3
H2A / H2B
H4
Coomassie
c
untagged-H3
His$_6$-H3
hHP1β
His$_6$-H3
H3
H2A / H2B
H4
Coomassie
d
untagged-H3
His$_6$-H3
mixed
untagged H3
hHP1β
His$_6$-H3
H3
H2A / H2B
H4
Coomassie
e
input
H3K9me0
H3K9$_C$me3
148 kDa
98 kDa
64 kDa
50 kDa
37 kDa
22 kDa
16 kDa
2x H3 - 2x HP1β
1x H3 - 2x HP1β
1x H3 - 1x HP1β
αHis$_6$
αH3
f
H3K9me0
H3K$_C$9me3
hHP1β I161A
hHP1β WT
NT,CD,HR(hHP1β)-GST
H3 + NT,CD,HR(hHP1β)-GST
H3 + hHP1β WT/I161A
free NT,CD,HR(hHP1β)-GST
free hHP1β WT/I161A
free H3
αH3
αHis$_6$

Supplementary Figure 6: Setup of the hHP1β/H3K9me3 chromatin photo-crosslinking experiment.

(**a**) Left, position of residue N57 (yellow) relative to the H3K9me3-peptide (blue) is shown in the model of the X-ray structure of the complex of the CD of CBX5 (hHP1α) and H3K9me3 (pdb: 3FDT). Right, position of residue Q158 is shown in the model of the X-ray structure of the CSD/CSD complex of CBX1 (hHP1β) (pdb:3Q6S). Using NCBI Cn3D viewer, amino acids that are within 4Å of the H3K9me3 peptide or the dimer interface surface were chosen as candidates for pBpa incorporation.

(**b**) Chromatin coprecipitation of hHP1β mutant proteins containing pBpa at position N57 (left) or at position N57 and Q158 (right) with oligonucleosomes. Precipitated material was run on SDS-PAGE and stained with Coomassie Blue. Input, 10 %.

(**c**) Chromatin coprecipitation of recombinant hHP1β WT with oligonucleosomes containing untagged- or His_6-H3. Precipitated material was run on SDS-PAGE and stained with Coomassie Blue. Input, 10 %.

(**d**) Input samples of the experiment shown in **Figure 4c** before photo-crosslinking were run on SDS-PAGE and stained with Coomassie Blue.

(**e**) Photo-crosslinking according to the scheme in **Figure 4b** was done with oligonucleosomes and His_6-hHP1β N57X Q158X. Samples were analyzed by western blotting using the indicated antibodies. Asterisks mark unspecific crosslinks.

(**f**) Photo-crosslinking according to the scheme in **Figure 4b** using the indicated proteins containing pBpa at position 57 of the CD(hHP1β) with untagged oligonucleosomes. Samples were analyzed by western blotting.

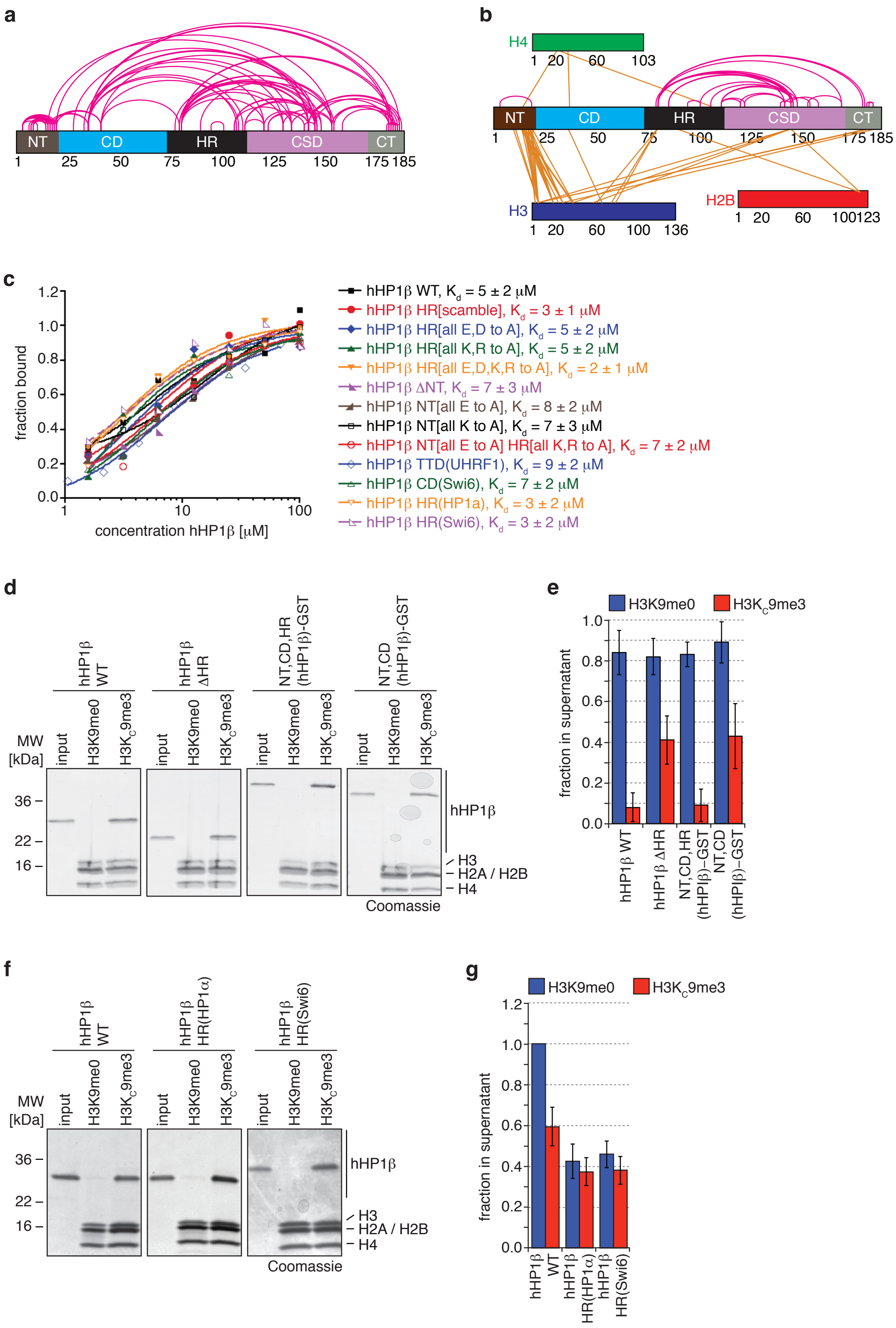

Supplementary Figure 7: Characterization of the chromatin effects of hHP1β proteins mutant in the NT and HR regions.

(a) Scheme representing EDC-crosslinks identified within the free hHP1β protein (2 μM) using mass spectrometry. For detailed listing of the crosslinks identified see **Supplementary Data 1**.

(b) Scheme representing EDC-crosslinks identified within the hHP1β/H3K9me3 oligonucleosomal array complex under conditions of chromatin coprecipitation (13.4 nM H3K9$_C$me3-oligonucleosomes, 2 μM

hHP1β) using mass spectrometry. For detailed listing of the identified crosslinks see **Supplementary Data 1**.
(**c**) Binding of the indicated hHP1β wild type (WT) and mutant proteins to a fluorescein-labeled H3K9me3-peptide was analyzed by FP at 150 mM NaCl. Apparent K_d deduced from the fitted curves are given.
(**d**) Chromatin coprecipitation of hHP1β WT and mutant proteins with oligonucleosomes. Precipitated material was run on SDS-PAGE and stained with Coomassie Blue. Input, 10 %.
(**e**) Oligonucleosomes at 2.5 nM were incubated with hHP1β wild type (WT) or mutant proteins at 5 μM and 150 mM NaCl (saturating conditions). DNA remaining in the supernatant after centrifugation was incubated with EtBr and measured by fluorescence reading. Data were normalized to DNA levels present in the input. Averages of three independent experiments are plotted; error bars represent s.d.; n = 3.
(**f**) Chromatin coprecipitation of hHP1β WT and mutant proteins with oligonucleosomes. Precipitated material was run on SDS-PAGE and stained with Coomassie Blue. Input, 10 %.
(**g**) Oligonucleosomes at 6.7 nM were incubated with hHP1β wild type (WT) or mutant proteins at 5 μM and 100 mM NaCl (non-saturating conditions). DNA remaining in the supernatant after centrifugation was incubated with EtBr and measured by fluorescence reading. Data were normalized to DNA levels present in the H3K9me0 chromatin/hHP1β WT sample. Averages of three independent experiments are plotted; error bars represent s.d.; n = 3.

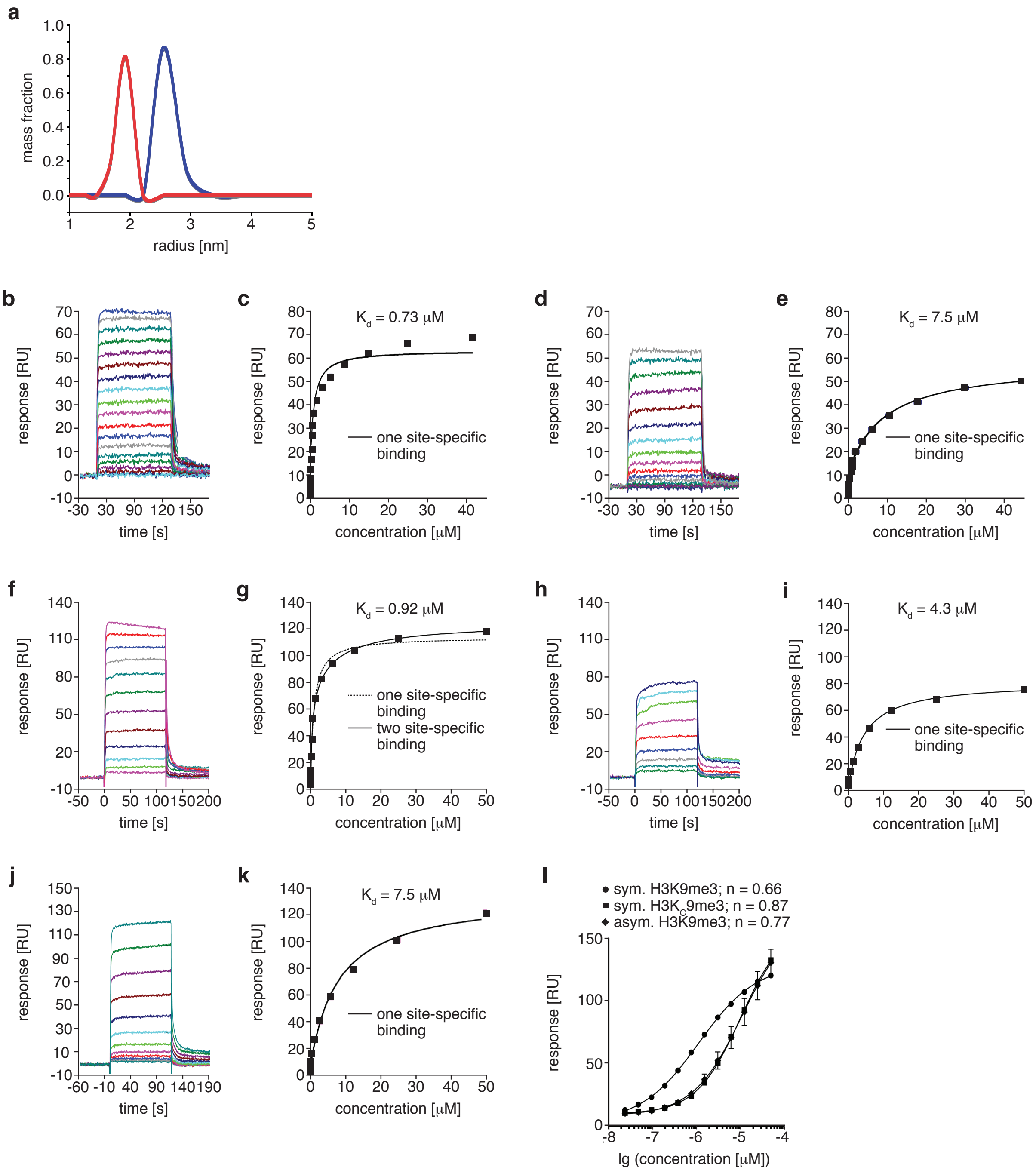

Supplementary Figure 8: hHP1β binds nucleosomes as dimer.

(**a**) Dynamic light scattering (DLS) analysis of recombinant hHP1β WT (blue) and I161A (red).

(**b**) SPR response traces of injection of hHP1β WT into Biacore cells containing biotinylated H3K9me3-peptide immobilized on a sensory chip via covalently coated streptavidin. Immobilized peptide was at 24 RU. Injection series are from 0.024 to 50 µM protein concentration.

(**c**) Blot of signal over concentration of the titration in (b) to deduce an apparent K_d value. Best fitting was achieved by one site-specific binding model.

(**d**) Same analysis as in (b) but with hHP1β I161A.

(**e**) Blot of signal over concentration of the titration in (d) to deduce an apparent K_d value. Best fitting was achieved by one site-specific binding model.

(**f**) SPR response traces of injection of hHP1β WT into Biacore cells containing biotinylated, symmetrically modified H3K9me3-mononucleosomes (i.e. both H3-tails carry H3K9me3) immobilized

on a sensory chip via covalently coated streptavidin. Immobilized mononucleosome was at 950 RU. Injection series are from 0.024 to 50 µM protein concentration.
(**g**) Blot of signal over concentration of the titration in (f) to deduce an apparent K_d value. For reference, fitting of the data to a one and two site-specific binding model is shown.
(**h**) Same analysis as in (f) but with hHP1β I161A.
(**i**) Blot of signal over concentration of the titration in (h) to deduce an apparent K_d value. Best fitting was achieved by one site-specific binding model.
(**j**) SPR response traces of injection of hHP1β WT into Biacore cells containing biotinylated, asymmetrically modified H3K9me3-mononucleosomes (i.e. only one H3 tail carries H3K9me3) immobilized on a sensory chip via covalently coated streptavidin. Immobilized mononucleosome was at 950 RU. Injection series are from 0.024 to 50 µM protein concentration.
(**k**) Blot of signal over concentration of the titration in (j) to deduce an apparent K_d value. Best fitting was achieved by one site-specific binding model.
(**l**) Hill plot of the SPR analysis of hHP1β WT interaction with symmetric and asymmetric H3K9me3-mononucleosomes. Hill coefficients (n) were deduced as slopes of the curves.

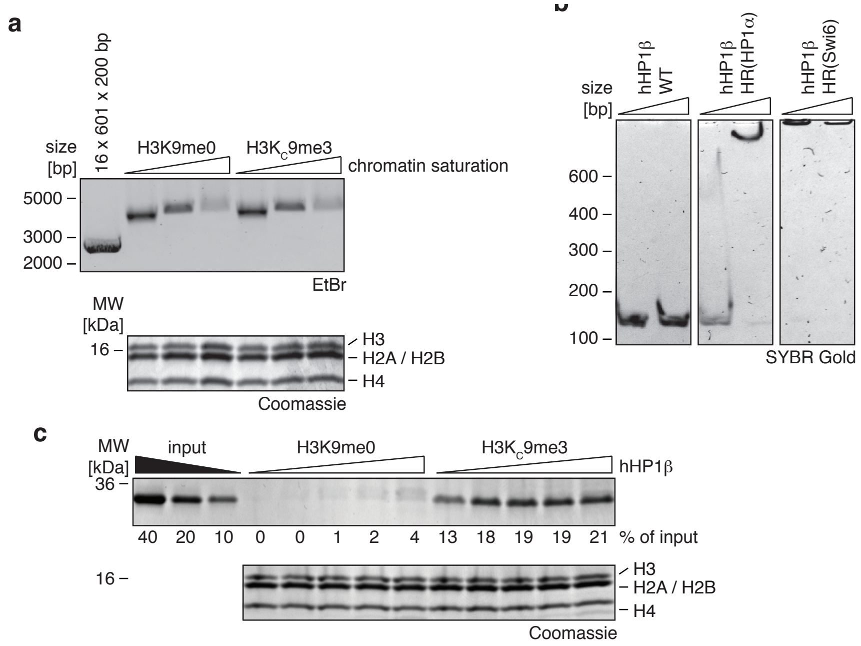

Supplementary Figure 9: Swi6 interacts with oligonucleosomes in a mode different from hHP1β.

(**a**) Oligonucleosomes reconstituted at 0.8 : 1.0, 1.0 : 1.0, and 1.2 : 1:0 octamer to positioning sequence ratio were analyzed on agarose gel stained with EtBr (top) or SDS-PAGE stained with Coomassie Blue (bottom).

(**b**) The indicated recombinant proteins were incubated with a DNA fragment of 150 bp at 500 : 1 and 1500 : 1 molar ratio. Complexes were separated by PAGE. DNA was stained with SYBR Gold.

(**c**) Chromatin coprecipitation of increasing concentrations of hHP1β WT (1 µM, 5 µM, 10 µM, 20 µM, 40 µM) with oligonucleosomes (6.7 nM). Precipitated material was run on SDS-PAGE and stained with Coomassie Blue. Intensity of bands was quantified in relation to the input. A representative experiment is shown.

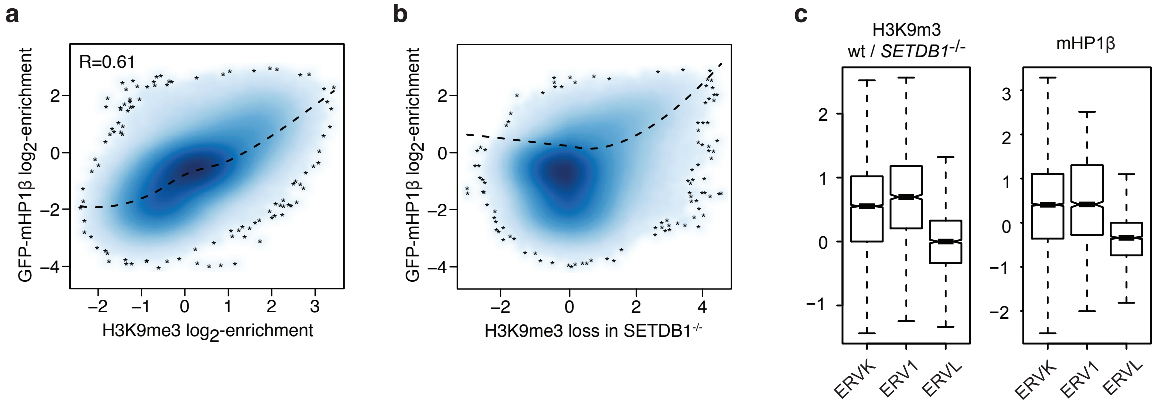

Supplementary Figure 10: **Genome-wide enrichment of mHP1β correlates with H3K9me3.**
(**a**) Plot of genome-wide correlation of GFP-mHP1β and H3K9me3 as deduced by ChIP-seq in mouse ESC and using 1kb-sized windows. Dashed line indicates the data trend computed by loess regression. (**b**) Analysis as in (a) but compared to loss of H3K9me3 in SETDB1$^{-/-}$ ESC. (**c**) Box plots showing mHP1β enrichment and SETDB1-dependent H3K9me3-levels at three different families of endogenous retroviral elements (ERV1, ERVK and ERVL). ERVL serves as negative control. Box indicates the inter-quartile range (IQR), while whiskers indicate 1.5 x of the IQR.

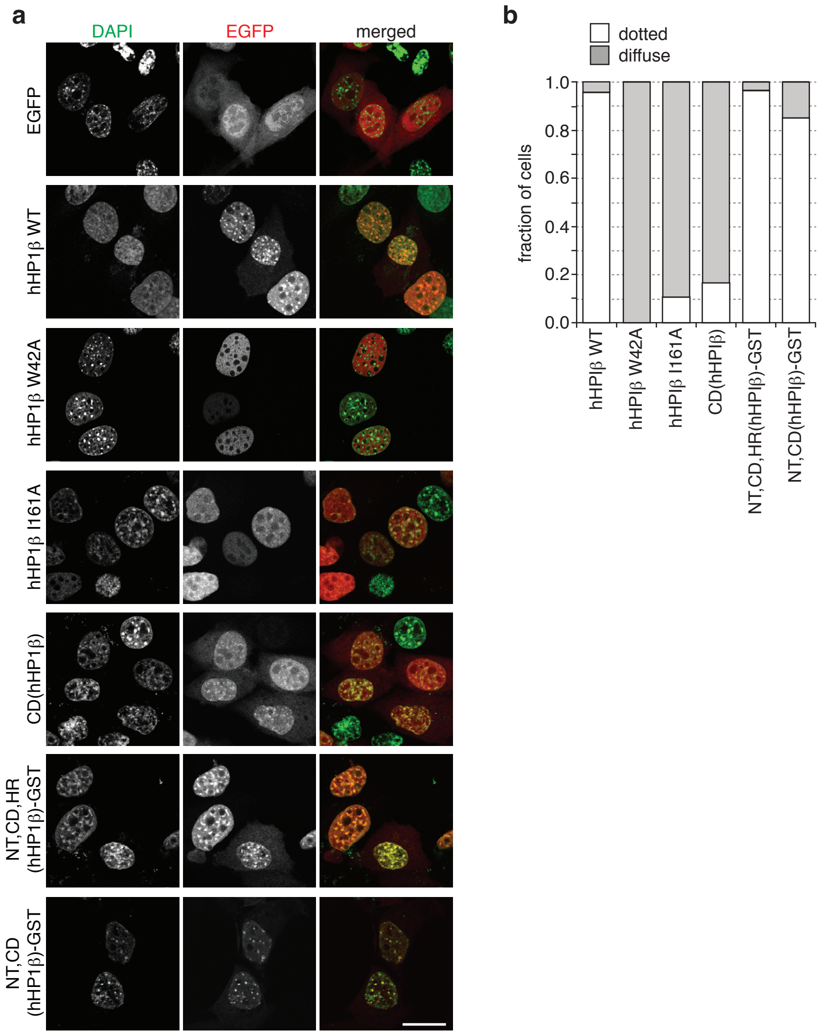

Supplementary Figure 11: Subnuclear distribution of hHP1β wild type and mutant proteins.

(**a**) The indicated proteins fused to EGFP were transiently expressed in mouse fibroblast cells. Representative fluorescence confocal images are shown. DNA was stained with DAPI. Scale bar corresponds to 25 µm.

(**b**) Quantification of the subnuclear distribution of the hHP1β WT and mutant proteins analyzed as in (a) by visual inspection. EGFP signal in each transfected cell was categorized into 'dotted' (see WT distribution in panel a) or 'diffuse' (see distribution of I161A in panel a). n >100 for each condition.

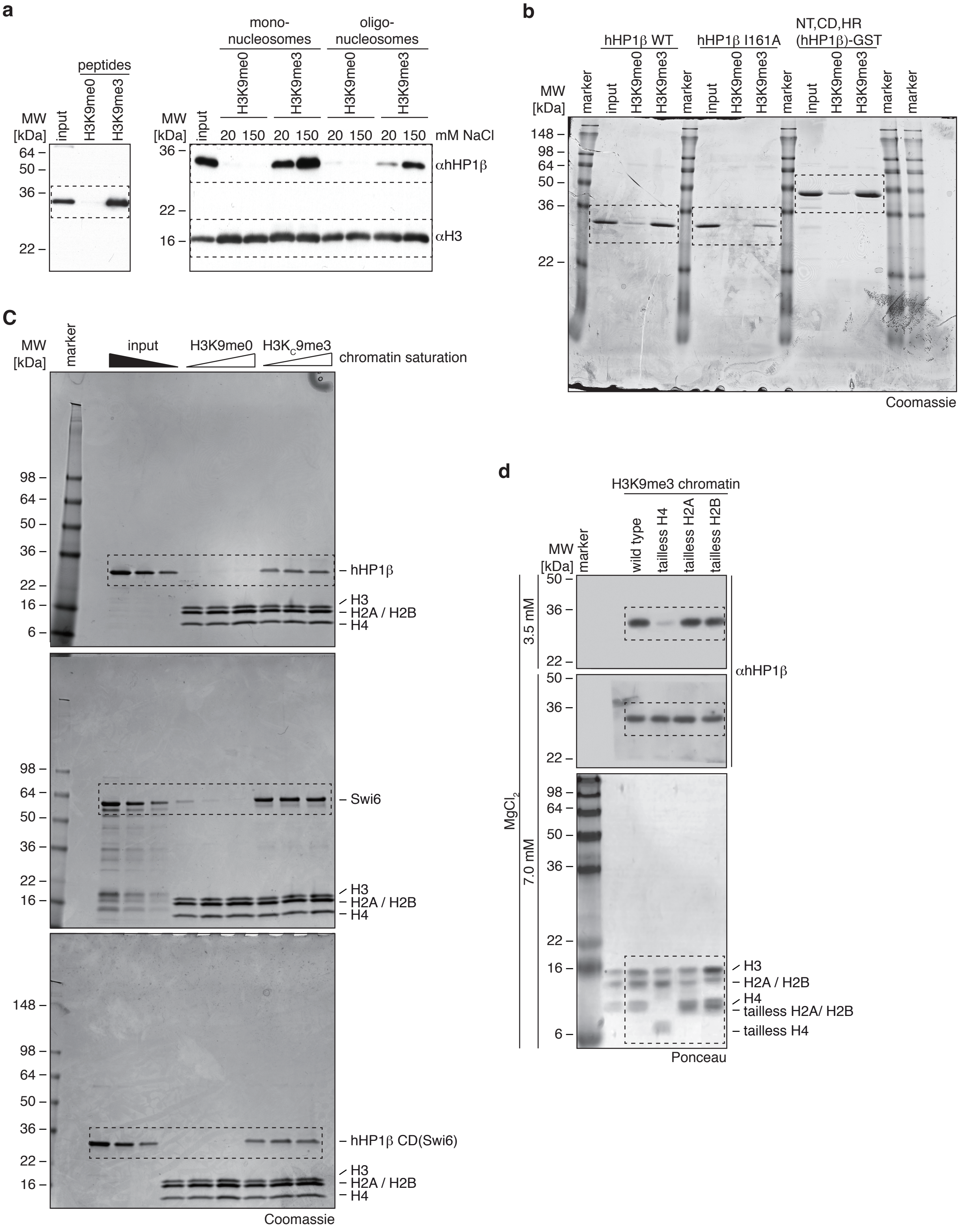

Supplementary Figure 12: Uncropped images of blots and gels of the main figures.
(**a**) Uncropped images corresponding to data shown in **Figure 1c**.
(**b**) Uncropped images corresponding to data shown in **Figure 2c**.
(**c**) Uncropped images corresponding to data shown in **Figure 3f**.
(**d**) Uncropped images corresponding to data shown in **Figure 5f**.
Areas of the blots and gels that are shown in the main figures are boxed (dashed lines).

SUPPLEMENTARY TABLES

Supplementary Table 1: Summary of conditions used in the different experiments of this study.

experiment	result	modification	template	Temp. (°C)	[NaCl] (mM)	[MgCl$_2$] (mM)
FP	Fig. S7c	H3K9me3	peptides	4	150	0
ITC	Tab.1; Fig. S4a	H3K9me3	peptides	20	20, 150	0
	Tab. 1	H3K9$_C$me3	peptides	20	20, 150	0
	Tab. 1	H3K9$_C$me3	mono-nucleosomes	20	20, 150	0
	Tab. 1; Fig. S4b	H3K9$_C$me3	oligo-nucleosomes	20	20, 150	0
SPR	Tab. 1; Fig. 2d; Fig. S2c,d; Fig. S8b-k	H3K9me3	peptide	4	150	0
	Tab. 1; Fig. S8i	H3K9me3	mono-nucleosomes	4	150	0
FCS	Fig. 3b	H3K9me3	oligo-nucleosomes	22[1]	150	0
SFM	Fig. 3g-h; Fig. S1a,b	H3K9me3	oligo-nucleosomes	4	50	0
	Fig. S1e	H3K9me3	oligo-nucleosomes	4	150	0
pull-down	Fig. 2c	H3K9me3	peptide	4	150	0
	Fig. 1c	H3K9me3	mono-nucleosomes	4	150	0
	Fig. 1c; Fig. 2b,e	H3K9me3	oligo-nucleosomes	4	150	0
chromatin coprecipitation	Fig. 1e,f; Fig. S1f-h; Fig. S2b	H3K9me3	oligo-nucleosomes	4	150	5
	Fig. 5d,f; Fig. S6b,c; Fig. S7d,f; Fig.S9c	H3K9$_C$me3	oligo-nucleosomes	4	150	5
	Fig. 3f	H3K9me3	oligo-nucleosomes	4	150	3.5, 7
chromatin sedimentation (single point)	Fig. 4a; Fig. S3b	H3K9me3	oligo-nucleosomes	4	150	0
	Fig. 5e; Fig. S7e,g	H3K9$_C$me3	oligo-nucleosomes	4	100	0
chromatin sedimentation (titration)	Fig. 3a	H3K9me3	oligo-nucleosomes	4	150	0

	Fig. 3e	H3K9me3	oligo-nucleosomes	4	150	0
	Fig. 3c	H3K9me3	oligo-nucleosomes	4	150	0
	Fig. 3d	H3K9me3	oligo-nucleosomes	4	25, 50, 100, 150	0
	Fig. S1d; Fig. S5	H3K9me3	oligo-nucleosomes	4	150	0.5 - 9.5[2]
UV crosslinking	Fig. 4c,d; Fig. S6e,f	$H3K9_Cme3$	oligo-nucleosomes	4	150	5[3]
chemical crosslinking	Fig. 5a; Fig. S7a,b	$H3K9_Cme3$	oligo-nucleosomes	RT	100	5[3]
EMSA	Fig. 1g; Fig. 5c; Fig. S9b	n//a		RT	100	5

[1]Preincubation of oligonucleosomes and hHP1β was performed at 4 °C. Measurements were carried out at the indicated temperature.

[2]in increments of 0.5 mM

[3]$MgCl_2$ was added at the end of incubation to coprecipitate oligonucleosomes and bound hHP1β.

Supplementary Table 2: Summary of pI values calculated for hHP1β wild type (WT) and mutant proteins.

pI values were obtained using the ExPASy ProtParam tool (http://web.expasy.org/protparam/). Green, chromatin aggregation behavior similar to wild type hHP1β; pink: loss of discrimination of H3K9me0 and H3K9me3; blue: compromised chromatin aggregation function.

	NT	CD	HR	NT,CD,HR	CSD,CT	total
hHP1α WT	4.66	6.79	9.82	8.85	4.71	5.71
hHP1γ WT	6.1	4.58	9.46	5.67	4.56	5.03
hHP1β WT	4.69	4.61	5.76	4.91	4.75	4.85
hHP1β HR[scramble]	4.69	4.61	5.80	4.91	4.75	4.86
hHP1β HR[all E,D to A]	4.69	4.61	11.56	9.08	4.75	6.24
hHP1β HR[all K,R to A]	4.69	4.61	3.46	4.23	4.75	4.39
hHP1β HR[all E,D,K,R to A]	4.69	4.61	6.40	4.73	4.75	4.74
hHP1β ΔNT	-	4.61	5.76	4.65	4.75	4.68
hHP1β NT[all E to A]	10.6	4.61	5.76	6.62	4.75	5.36
hHP1β NT[all K to A]	3.36	4.61	5.76	4.57	4.75	4.62
hHP1β NT[all E to A] HR[all K,R to A]	10.6	4.61	3.46	4.46	4.75	4.56
hHP1β HR(hHP1α)	4.69	4.61	9.82	5.47	4.75	5.13
hHP1β HR(Swi6)	4.69	4.61	8.94	5.24	4.75	5.06
hHP1β CD(Swi6)	4.69	5.17	5.76	5.14	4.75	4.99
hHP1β TTD(UHRF1)	4.69	4.86	5.76	4.97	4.75	4.91

Supplementary Table 3: List of plasmids used in this study.
Further details of cloning procedures are available upon request.

**cDNA**	**backbone**	**expression**	**comments**
hHP1β WT	pET11a	*E.coli*	
hHP1β W42A	pET11a	*E.coli*	
hHP1β I161A	pET11a	*E.coli*	
CD(hHP1β)	pET11a	*E.coli*	
CSD(hHP1β)	pET11a	*E.coli*	
hHP1β ΔHR	pET11a	*E.coli*	
NT,CD,HR(hHP1β)-GST	pET11a	*E.coli*	
CD(hHP1β)-GST	pET11a	*E.coli*	
hHP1α WT	pColdI	*E.coli*	
hHP1β WT	pColdI	*E.coli*	
hHP1β HR[scramble]	pColdI	*E.coli*	synthetic cDNA, codon optimized
hHP1β HR[all E,D,K,R to A]	pColdI	*E.coli*	synthetic cDNA, codon optimized
hHP1β HR[all E,D to A]	pColdI	*E.coli*	synthetic cDNA, codon optimized
hHP1β HR[all K,R to A]	pColdI	*E.coli*	synthetic cDNA, codon optimized
hHP1β HR(hHP1α)	pColdI	*E.coli*	synthetic cDNA, codon optimized
hHP1β HR(Swi6)	pColdI	*E.coli*	synthetic cDNA, codon optimized
hHP1β ΔNT	pColdI	*E.coli*	
hHP1β NT[all K to A]	pColdI	*E.coli*	synthetic cDNA, codon optimized
hHP1β NT[all E to A]	pColdI	*E.coli*	synthetic cDNA, codon optimized
hHP1β NT[all K to A] HR[all E,D to A]	pColdI	*E.coli*	synthetic cDNA, codon optimized
hHP1β NT[all E to A] HR[all K,R to A]	pColdI	*E.coli*	synthetic cDNA, codon optimized
hHP1β TTD(UHRF1)	pColdI	*E.coli*	synthetic cDNA, codon optimized
hHP1β CD(Swi6)	pColdI	*E.coli*	synthetic cDNA, codon optimized
hHP1β WT	pCDF1	*E.coli*	
hHP1β N57X	pCDF1	*E.coli*	'amber' stop codon at position 57 for pBpa incorporation
hHP1β N57X I161A	pCDF1	*E.coli*	amber' stop codon at position 57 for pBpa incorporation
NT,CD,HR(hHP1β)-GST N57X	pCDF1	*E.coli*	amber' stop codon at position 57 for pBpa incorporation
hHP1β N57X Q158X	pCDF1	*E.coli*	amber' stop codon at positions 57 and 158 for pBpa incorporation

-	pYFP-LacI-NSL C1	mammalian cells	targeting to LacO transgene
hHP1β WT	pYFP-LacI C1	mammalian cells	targeting to LacO transgene
hHP1β W42A	pYFP-LacI C1	mammalian cells	targeting to LacO transgene
hHP1β I161A	pYFP-LacI C1	mammalian cells	targeting to LacO transgene
NT,CD,HR(hHP1β)-GST	pYFP-LacI C1	mammalian cells	targeting to LacO transgene
hHP1β ΔHR	pYFP-LacI C1	mammalian cells	targeting to LacO transgene

SUPPLEMENTARY NOTE 1

The binding events analyzed on peptides and mononucleosomes are consistent with a bivalent hHP1β dimer interacting with H3K9me3 on the Bicacore chip surface (Supplementary figure 8). The deviation from one-site specific binding observed for hHP1β WT is caused by the apparent increase of local concentration of hHP1β after binding of the first molecule of the dimer.[1] The second molecule of the dimer then has a higher likelihood of finding an H3K9me3-target, either in the form of another immobilized histone tail peptide or another H3-tail in the context of a symmetrically modified mononucleosome. This is enabled by the flexibility and reach of the extended conformation of the hHP1β dimer.[2] In agreement, the binding of the monomeric hHP1β I161A mutant protein is fully consistent with one-site specific interaction. There is no evidence of a predetermined or induced interaction of the CD with itself as suggested for Swi6.[3] In such case additional protein would be recruited after initial binding of hHP1β to the surface in the case of dimeric WT as well as monomeric I161A proteins. This would be reflected in significant deviation from one site-specific binding. Also, the data of interaction of hHP1β with an asymmetrically modified H3K9me0/H3K9me3-mononucleosome fit better to a one site-specific binding mode compared to the interaction with a symmetrically modified H3K9me3/H3K9me3-mononucleosome. Note that the nucleosome is 100x larger than the immobilized peptide thereby causing a much larger response in the SPR readings. Therefore, the total concentration of H3-tail immobilized at 24 RU of peptide is actually higher than for mononucleosomes at 950 RU. If further taking into account that streptavidin itself is a tetramer, the display of H3K9me3-interaction surface in both scenarios – immobilized peptides and mononucleosomes – is similar. While dimeric hHP1β can find two H3K9me3-tail interaction partners with peptides in close proximity or within a single, symmetrically modified H3K9me3-mononucleosome, such interaction is limited in the case of asymmetrically modified mononucleosomes.

Our interpretation is congruent with all experimental schemes yielding similar SPR readings at the highest concentration of hHP1β injected. Also apparent binding is stronger in case of bivalent interaction mode (i.e. hHP1β WT with peptide or symmetrically modified mononuclesomes) as compared to inter-action of only one CD (i.e. monomeric hHP1β I161A with peptide or symmetrically modified mononuc-leosomes or hHP1β WT on asymmetrically modified mononucleosomes). The CSD-CSD interaction for dimerization of hHP1β monomers is in the nanomolar regime (as deduced by ITC dilution experiments, not shown). It is therefore three orders of magnitude stronger than the binding of the CD to H3K9me3. Our SPR titration experiments started with 24 nM hHP1β protein and we therefore do not expect any significant contribution of the hHP1β-dimerization equilibrium to the SPR signals.

SUPPLEMENTARY METHODS

Analytical ultracentrifugation

Sedimentation velocity analysis was performed on an Optima XL-A analytical ultracentrifuge (Beckman-Coulter, Krefled, Germany) using an An60Ti rotor and double sector cells (path length 12 mm). 400 μl samples at an OD_{260nm} of 0.3-1 in TEA buffer were analyzed at 0 °C and a rotor speed of 35,000 rpm. The concentration profile was recorded by UV measurement at 260 nm. Buffer density, viscosity and protein partial specific volumes were calculated using the Sednterp software (http://www.rasmb.bbri.org/). Raw data was analyzed using the program SEDFIT (version 11.8).[4] For each analysis 30 scans were continuously collected. The size distributions were calculated while floating meniscus, frictional coefficient and baseline, but keeping the buffer parameters constant, with a confidence level of $P = 0.68$, a resolution of $n = 150$, and sedimentation coefficients between 2 and 40 S.

SUPPLEMENTARY REFERENCES

1. Marquart, J.A. SPR Theory and Practice (2013).
2. Munari, F. et al. Methylation of lysine 9 in histone H3 directs alternative modes of highly dynamic interaction of heterochromatin protein hHP1beta with the nucleosome. *J Biol Chem* **287**, 33756-65 (2012).
3. Canzio, D. et al. Chromodomain-mediated oligomerization of HP1 suggests a nucleosome-bridging mechanism for heterochromatin assembly. *Mol Cell* **41**, 67-81 (2011).
4. Schuck, P. A model for sedimentation in inhomogeneous media. I. Dynamic density gradients from sedimenting co-solutes. *Biophys Chem* **108**, 187-200 (2004).
